# Ras promotes macropinocytic nutrient uptake by suppressing the albumin recycling receptor FcRn

**DOI:** 10.1038/s44319-026-00787-4

**Published:** 2026-04-29

**Authors:** Rafael Paschoal de Campos, Xuxia Wu, Aslihan Inal, Zhao Liu, Craig B Thompson, Wilhelm Palm

**Affiliations:** 1 Division of Cell Signaling and Metabolism, German Cancer Research Center (DKFZ) and DKFZ-ZMBH Alliance, Heidelberg, 69120 Germany; 2 Faculty of Biosciences, University of Heidelberg, Heidelberg, 69120 Germany; 3 Medical Faculty Heidelberg, University of Heidelberg, Heidelberg, 69120 Germany; 4 Cancer Biology and Genetics Program, Memorial Sloan Kettering Cancer Center, New York, NY 10065 USA; 5 Present Address: Institute of Pathology, University Hospital Heidelberg, Heidelberg University, Heidelberg, 69120 Germany

**Keywords:** Cancer, Metabolism, Signal Transduction

## Abstract

Macropinocytosis and lysosomal degradation of extracellular protein constitute a nutrient acquisition pathway in Ras-driven cancers. By catabolizing albumin, the most abundant plasma protein, Ras-transformed cells sustain growth in environments where free amino acids are scarce. Under physiological conditions, however, albumin is normally protected from lysosomal degradation by the neonatal Fc receptor (FcRn), which recycles albumin back to the extracellular space. Here, by investigating how cancer cells overcome FcRn-mediated albumin recycling, we identify the Ras–Erk MAPK signaling pathway as a critical regulator of FcRn. Expression of constitutively active Ras variants or stimulation with growth factors represses FcRn transcription through activation of the MAPK pathway, leading to decreased FcRn protein abundance. Conversely, pharmacological inhibition of Ras–MAPK signaling de-represses FcRn expression. Restoring FcRn levels in Ras-transformed cells limits lysosomal albumin degradation and impairs the proliferation of cells that depend on albumin as an essential amino acid source. Thus, oncogenic Ras signaling promotes the nutritional utilization of albumin by suppressing FcRn, thereby supporting cancer cell adaptation to nutrient-poor environments.

## Synopsis

Oncogenic Ras signaling suppresses the endosomal albumin recycling receptor, FcRn, thereby promoting the nutritional utilization of albumin and supporting cancer cell adaptation to amino acid-poor environments.

- Cancer cells with oncogenic activation of the Ras–Erk MAPK pathway exhibit reduced FcRn protein abundance.
- Ras–Erk MAPK signaling suppresses FcRn transcription by downregulating its promoter activity.
- Restoring FcRn expression limits cellular accumulation and lysosomal degradation of albumin, suppressing proliferation of Ras-transformed cells that depend on albumin as a nutrient source.

## Abstract (web summary)

Oncogenic Ras signaling suppresses the endosomal albumin recycling receptor, FcRn, thereby promoting the nutritional utilization of albumin and supporting cancer cell adaptation to amino acid-poor environments.

## Graphical abstract

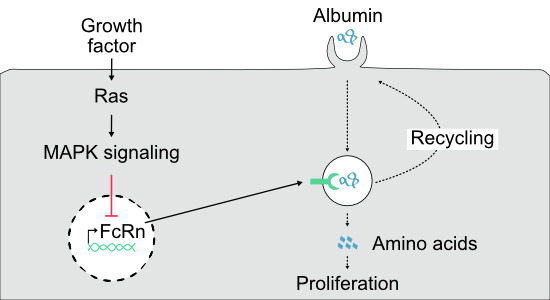

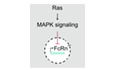

## Introduction

Activating mutations in Ras GTPases (Hras, Kras and Nras) are among the most frequent oncogenic events in human cancers. Whereas Ras is normally activated by upstream growth factor–receptor tyrosine kinase signaling, cancer-associated mutations interfere with its ability to hydrolyze GTP, thus locking Ras into the GTP-bound, constitutively active state (Hobbs et al, 2016; Prior et al, 2020; Simanshu et al, 2017). Oncogenic Ras drives signaling through multiple effector pathways, in particular the Raf–Mek–Erk mitogen-activated protein kinase (MAPK) and phosphoinositide 3-kinase (PI3-kinase) pathways, which promote cancer cell survival and growth. A hallmark of Ras-driven carcinogenesis is the dysregulation of cellular metabolism (Kerk et al, 2021; Palm and Thompson, 2017). One such metabolic program is the utilization of extracellular proteins as a nutrient source. By inducing macropinocytosis—a non-selective endocytic pathway—Ras enables cells to internalize large quantities of extracellular proteins, which can subsequently be degraded in lysosomes to release their amino acid content (Commisso et al, 2013; Kamphorst et al, 2015; Palm et al, 2015). By unlocking the abundant nutrient stores of extracellular proteins, Ras thus enhances the metabolic flexibility of cancer cells and supports their growth in nutrient-poor tumors.

Albumin, the most abundant protein in circulation, is the best-characterized macropinocytic nutrient in cancer. In vitro, supplementing culture media with physiological concentrations of albumin enables Ras-transformed cells to proliferate under conditions in which free amino acids are limiting (Commisso et al, 2013; Kamphorst et al, 2015; King et al, 2020; Lee et al, 2019; Palm et al, 2017; Palm et al, 2015). In vivo, enhanced uptake and lysosomal catabolism of albumin have been documented in pancreatic cancer, a Kras-driven, nutrient-poor tumor type that exploits macropinocytic nutrient acquisition (Davidson et al, 2017; Kamphorst et al, 2015). Accordingly, pharmacological and genetic manipulations that inhibit macropinocytosis or lysosomal protein degradation stall the growth of cancer cells that depend on albumin in cell culture and exhibit tumor-suppressive effects in mouse models of Ras-driven cancers (Commisso et al, 2013; Lambies et al, 2024; Palm et al, 2015; Pechincha et al, 2022).

The efficient albumin catabolism observed in Ras-transformed cells presents a paradox: under physiological conditions, albumin is normally protected from lysosomal degradation by the neonatal Fc receptor (FcRn). FcRn is a major histocompatibility complex I (MHC-I)-related receptor, consisting of a heavy α chain encoded by the *FCGRT* gene, hereafter referred to as FcRn, and β2 microglobulin encoded by the *B2M* gene, a shared subunit of MHC-I-related complexes (Pyzik et al, 2019; Sand et al, 2014). FcRn binds internalized albumin at the acidic pH of endosomes and transports albumin back to the cell surface for release into the extracellular space, thus preventing its lysosomal degradation. FcRn plays a key role in systemic albumin homeostasis. In FcRn-deficient mice, the failure to salvage albumin results in increased cellular turnover and decreased plasma concentrations (Chaudhury et al, 2003; Kim et al, 2006). In cancer, the relevance of FcRn remains unclear, but several studies have observed reduced FcRn expression in tumors, which was associated with increased tumor growth, poor prognosis or improved efficacy of albumin-bound therapeutics (Dalloneau et al, 2016; Liu et al, 2019; Swiercz et al, 2017). The molecular mechanisms regulating FcRn expression in cancer cells, however, have been unknown.

Here, we demonstrate that FcRn expression is suppressed by Ras. Cancer-associated Ras variants downregulate FcRn transcription through the Raf–Mek–Erk MAPK signaling pathway, leading to a decline in FcRn protein abundance. Restoring FcRn expression in Ras-transformed cells limits lysosomal albumin degradation and impairs cell proliferation that depends on albumin as a nutrient. Thus, FcRn suppression constitutes a mechanism by which Ras promotes cancer cell adaptation to amino acid deprivation.

## Results and discussion

### FcRn expression is suppressed by Ras

**Figure 1:**
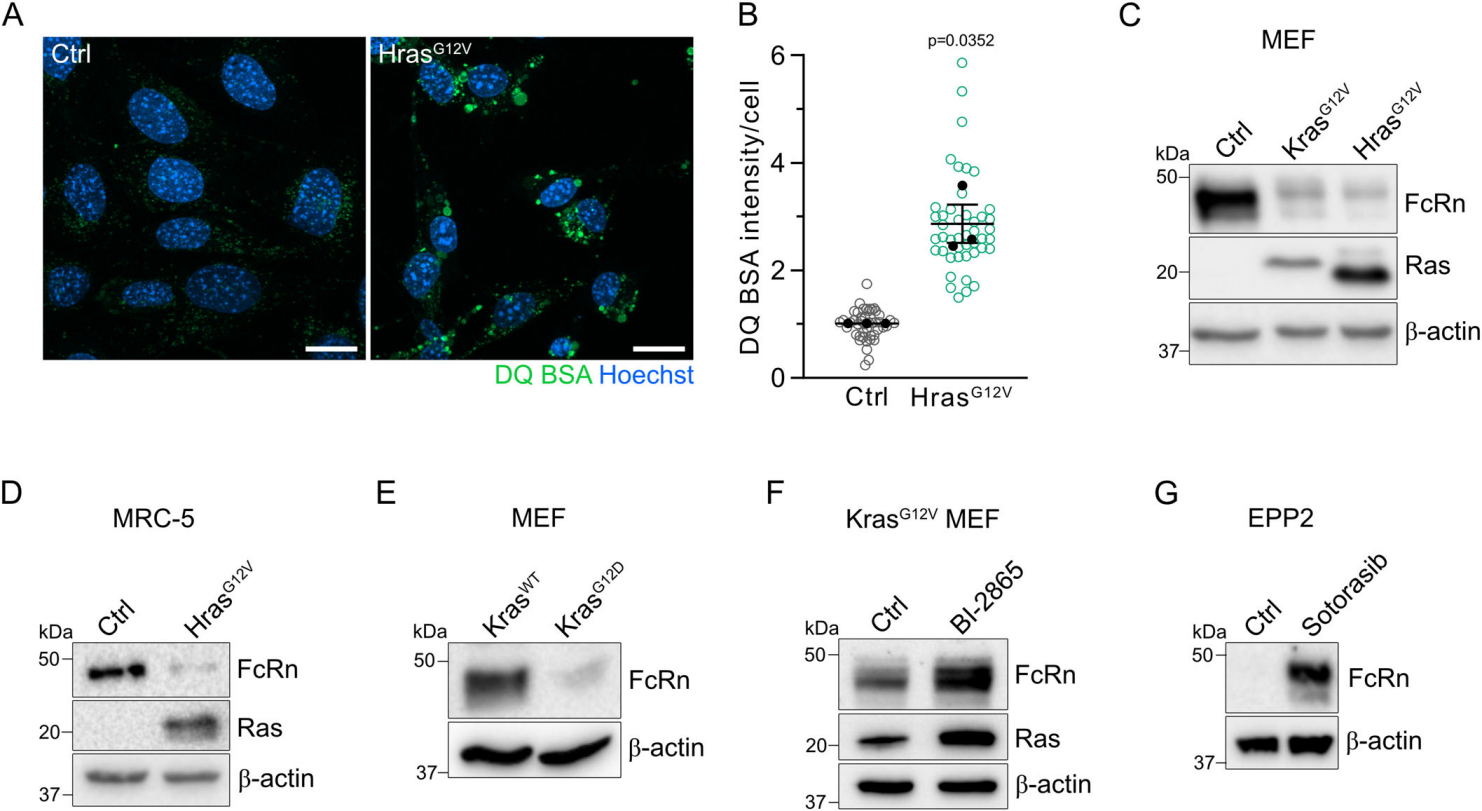
The albumin recycling receptor FcRn is suppressed by oncogenic Ras. (**A**) DQ BSA degradation in MEFs after 16 h Hras^G12V^ induction and 5 h DQ BSA uptake, analyzed by microscopy. Scale bars = 20 µm. (**B**) Quantification of DQ BSA fluorescence of cells shown in (**A**). (**C**–**G**) FcRn protein abundance, analyzed by immunoblotting; (**C**) MEFs after 16 h Kras^G12V^ or Hras^G12V^ induction; (**D**) MRC-5 fibroblasts expressing Hras^G12V^; (**E**) MEFs harboring endogenous Kras^G12D^; (**F**) MEFs after 16 h Kras^G12V^ induction and treatment with the pan-Kras inhibitor BI-2865 [2 µM]; (**G**) murine pancreatic cancer EPP2 cells harboring endogenous Kras^G12C^ after 16 h treatment with the Kras^G12C^-selective inhibitor sotorasib [0.1 µM]. All experiments were performed in 0.1% FBS. Data information: (**B**) Data were normalized replicate mean ± SEM (closed circles; *n* = 3 independent experiments) and fields of view (open circles; 15 per replicate). *p* values were calculated using a two-tailed unpaired *t*-test with Welch correction. (**C**–**G**) Representative data from one out of *n* = 3 independent experiments. Source data are available online for this figure.

**Figure EV1:**
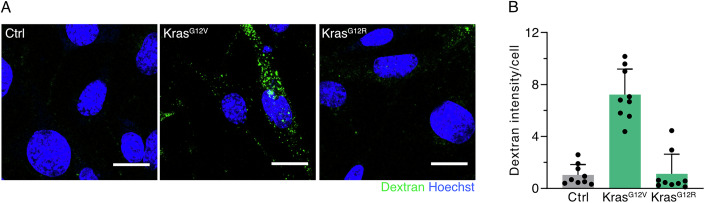
Macropinocytosis induction by Kras^G12V^ but not by atypical Kras^G12R^. (**A**) Intracellular levels of 70 kDa dextran in MEFs after 16 h induction of Kras^G12V^ or Kras^G12R^ and 1 h dextran uptake. Scale bars = 20 µm. (**B**) Quantification of dextran fluorescence of cells shown in A. Experiments were performed in 0.1% FBS. Data information: (**B**) Data are mean ± SD (*n* = 9 fields of view from one representative out of *n* = 3 independent experiments). Source data are available online for this figure.

To understand why Ras-transformed cells efficiently take up and degrade albumin, we investigated whether Ras signaling regulates the albumin recycling receptor, FcRn. We first used immortalized mouse embryonic fibroblasts (MEFs) as a genetically defined system in which to study Ras signaling. MEFs were stably transduced with constitutively active Ras variants under a doxycycline-inducible promoter. Hras^G12V^ expression was induced by adding doxycycline to the culture medium for 16 h, and exogenous growth factor stimuli were removed by lowering fetal bovine serum (FBS) concentrations to 0.1%. We then examined intracellular albumin catabolism using DQ BSA, a self-quenched albumin probe that emits fluorescence upon lysosomal degradation (Palm et al, 2015). Cells were incubated with DQ BSA for 5 h, and lysosomal DQ BSA degradation was quantified by live-cell microscopy. The induction of Hras^G12V^ expression led to a significant increase in intracellular albumin degradation (Fig. 1A,B). Macropinocytosis was also strongly increased upon induction of Kras^G12V^ (Fig. EV1A,B), consistent with previous results (Commisso et al, 2013; Palm et al, 2015). Next, we determined the impact of Ras signaling on FcRn protein abundance by immunoblotting. In MEFs, induction of Kras^G12V^ or Hras^G12V^ expression strongly reduced FcRn protein levels (Fig. 1C). A similar reduction of FcRn abundance was observed in human MRC-5 fibroblasts stably expressing Hras^G12V^ (Fig. 1D) and in MEFs harboring an endogenous Kras^G12D^ allele (Fig. 1E). Conversely, pharmacological inhibition of Ras signaling strongly increased FcRn levels, both in Kras^G12V^-expressing MEFs treated with the pan-Kras inhibitor BI-2865 (Kim et al, 2023) and in pancreatic cancer EPP2 cells (harboring Kras^G12C^) treated with the Kras^G12C^-selective inhibitor sotorasib (Canon et al, 2019) (Fig. 1F,G). Thus, constitutive activation of Ras signaling leads to the downregulation of FcRn protein.

**Figure 2:**
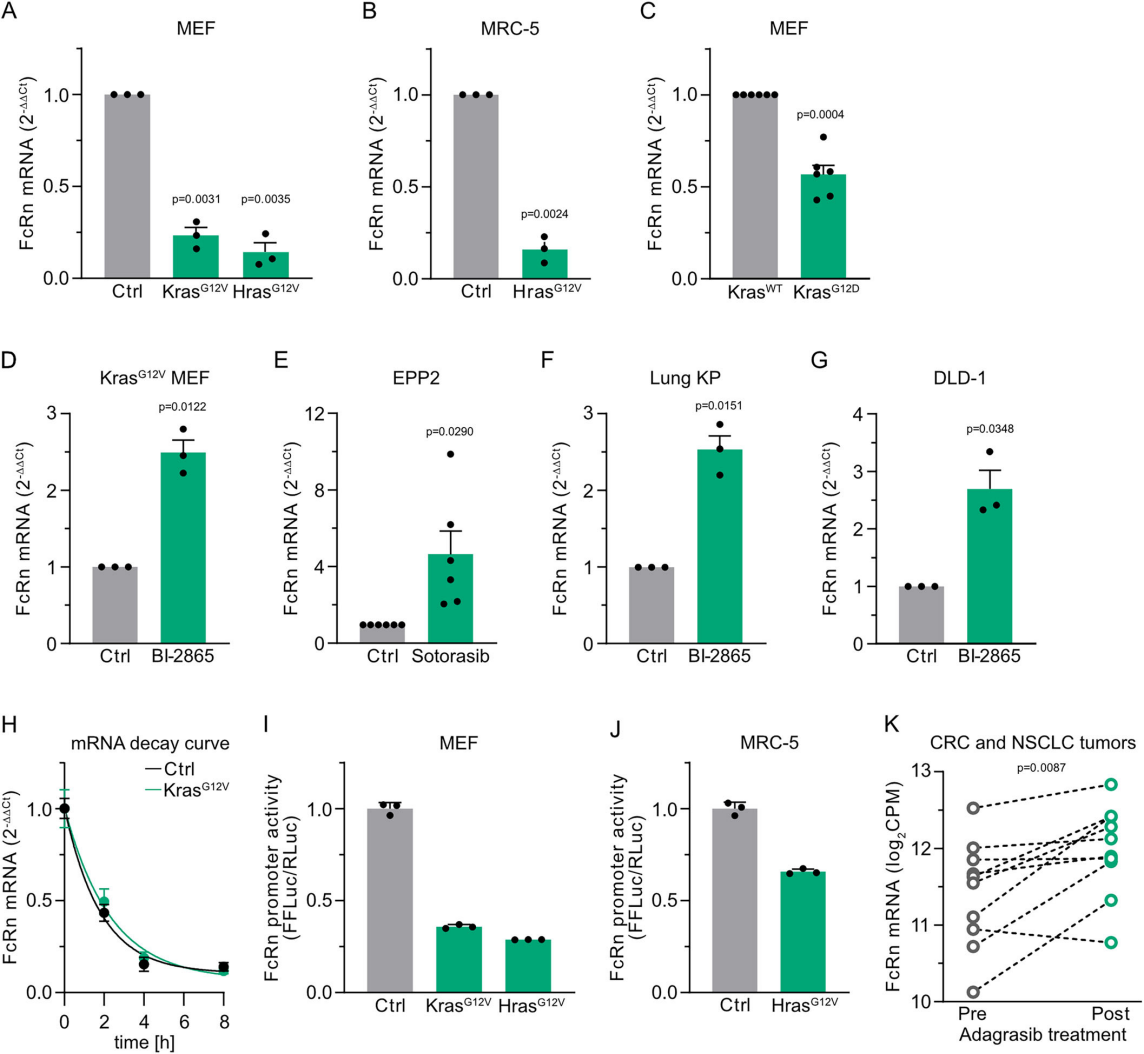
FcRn is suppressed by Ras at the transcriptional level. (**A**–**C**) Relative FcRn mRNA abundance, analyzed by RT-qPCR; (**A**) MEFs after 16 h Kras^G12V^ or Hras^G12V^ induction; (**B**) MRC-5 fibroblasts expressing Hras^G12V^; (**C**) MEFs harboring endogenous Kras^G12D^. (**D**–**G**) Relative FcRn mRNA abundance in Kras-mutant cells treated for 16 – 24 h with indicated Kras inhibitors, analyzed by RT-qPCR; (**D**) MEFs after 16 h Kras^G12V^ induction, BI-2865 [2 µM]; (**E**) murine pancreatic cancer EPP2 cells harboring Kras^G12C^, sotorasib [0.1 µM]; (**F**) lung cancer KP cells harboring Kras^G12D^, BI-2865 [5 µM]; (**G**) colorectal cancer DLD-1 cells harboring Kras^G13D^, BI-2865 [5 µM]. (**H**) Relative FcRn mRNA abundance in MEFs after induction of Kras^G12V^ and treatment with the transcription inhibitor actinomycin D [5 µg/ml] for the indicated time periods, analyzed by RT-qPCR. (**I**,** J**) Relative FcRn promoter activity, analyzed with dual-luciferase reporter assay (Firefly luciferase: FFLuc; Renilla luciferase: RLuc); (**I**) MEFs after 16 h induction of Kras^G12V^ or Hras^G12V^; (**J**) MRC-5 fibroblasts expressing Hras^G12V^. (**K**) FcRn levels from colorectal (CRC) and non-small cell lung cancer (NSCLC) patients before and 8 days after treatment with the Kras^G12C^-selective inhibitor adagrasib, analyzed by RNA-seq (Klomp et al, 2024). Cell culture experiments were performed in 0.1% FBS. Data information: (**A**–**G**) Data are normalized replicate mean ± SEM (**A**, **B**, **D**, **F**, **G** *n* = 3; **C**, **E** *n* = 6 independent experiments). *p* values were calculated using a two-tailed unpaired *t*-test with Welch correction. (**H**) Data are mean ± SD (three technical replicates from one representative out of *n* = 3 independent experiments). (**I**,** J**) Data are mean ± SD (three technical replicates from one representative out of *n* = 2–3 independent experiments). (**K**) Paired data of *n* = 10 patients; *p* values were calculated using a two-tailed paired *t*-test. Source data are available online for this figure.

To further investigate how FcRn is regulated by Ras, we examined changes in FcRn transcripts using RT-qPCR. The abundance of FcRn mRNA was strongly decreased in the various cell lines expressing constitutively active Ras: MEFs following the induction of Kras^G12V^ or Hras^G12V^ (Fig. 2A), MRC-5 fibroblasts transformed with Hras^G12V^ (Fig. 2B) and Kras^G12D^ MEFs (Fig. 2C). Consistently, pharmacological Kras inhibition restored FcRn mRNA abundance in Kras^G12V^ MEFs and in several cancer cell lines harboring oncogenic Kras mutations (Fig. 2D–G).

**Figure EV2:**
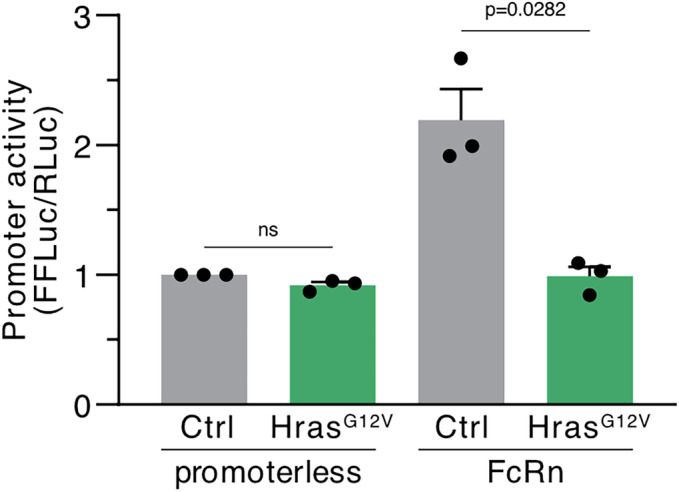
The FcRn promoter is regulated by Ras signaling. Relative luciferase expression in MEFs after 16 h induction of Hras^G12V^, analyzed with dual-luciferase reporter assay (Firefly luciferase: FFLuc; Renilla luciferase: RLuc). Firefly luciferase was stably expressed in a basal or FcRn-regulated manner by transducing cells with Lenti-luciferase-P2A-Neo without promoter (promoterless) or under control of the FcRn promoter, followed by G418 (Neo) selection. Experiments were performed in 0.1% FBS. Data information: Data are normalized replicate mean ± SEM (*n* = 3 independent experiments with 3 technical replicates). *p* values were calculated using a two-tailed unpaired *t*-test with Welch correction (ns not significant). Source data are available online for this figure.

We reasoned that Ras signaling could lower the abundance of FcRn transcripts by either decreasing mRNA stability or suppressing promoter activity. To examine mRNA stability, we inhibited transcription with actinomycin D and quantified FcRn mRNA abundance over time. FcRn mRNA decayed at comparable rates in MEFs expressing Kras^G12V^ and control cells (Fig. 2H). Thus, Ras does not affect FcRn transcript stability. To investigate whether Ras regulated FcRn promoter activity, we used a dual-luciferase system. We generated Firefly luciferase constructs expressed under the control of the murine or human FcRn promoters. We then stably transduced cells with the Firefly luciferase FcRn reporter and, to normalize for overall gene expression, with constitutively expressed Renilla luciferase. Firefly luciferase signal was substantially decreased in MEFs following the induction of Kras^G12V^ or Hras^G12V^, indicating reduced activity of the murine FcRn promoter (Fig. 2I). Similarly, Firefly luciferase expression from the human FcRn promoter was decreased in Hras^G12V^–transformed MRC-5 fibroblasts (Fig. 2J). By contrast, a promoterless luciferase construct remained unresponsive to Hras^G12V^ expression (Fig. EV2). These results suggest that constitutively active Ras downregulates FcRn expression by suppressing FcRn promoter activity.

The above findings demonstrate that cancer-associated Ras mutations potently repress FcRn transcription in vitro. To examine whether these changes are observed in naturally occurring tumors, we took advantage of available gene expression data from tumor biopsies of patients with Kras^G12C^-driven colorectal or non-small cell lung cancer before and after treatment with the Kras^G12C^-selective inhibitor adagrasib (Klomp et al, 2024). FcRn mRNA abundance was significantly increased in tumors in this patient cohort following adagrasib treatment (Fig. 2K).

### Ras suppresses FcRn through the Raf–Mek–Erk MAPK signaling pathway

**Figure 3:**
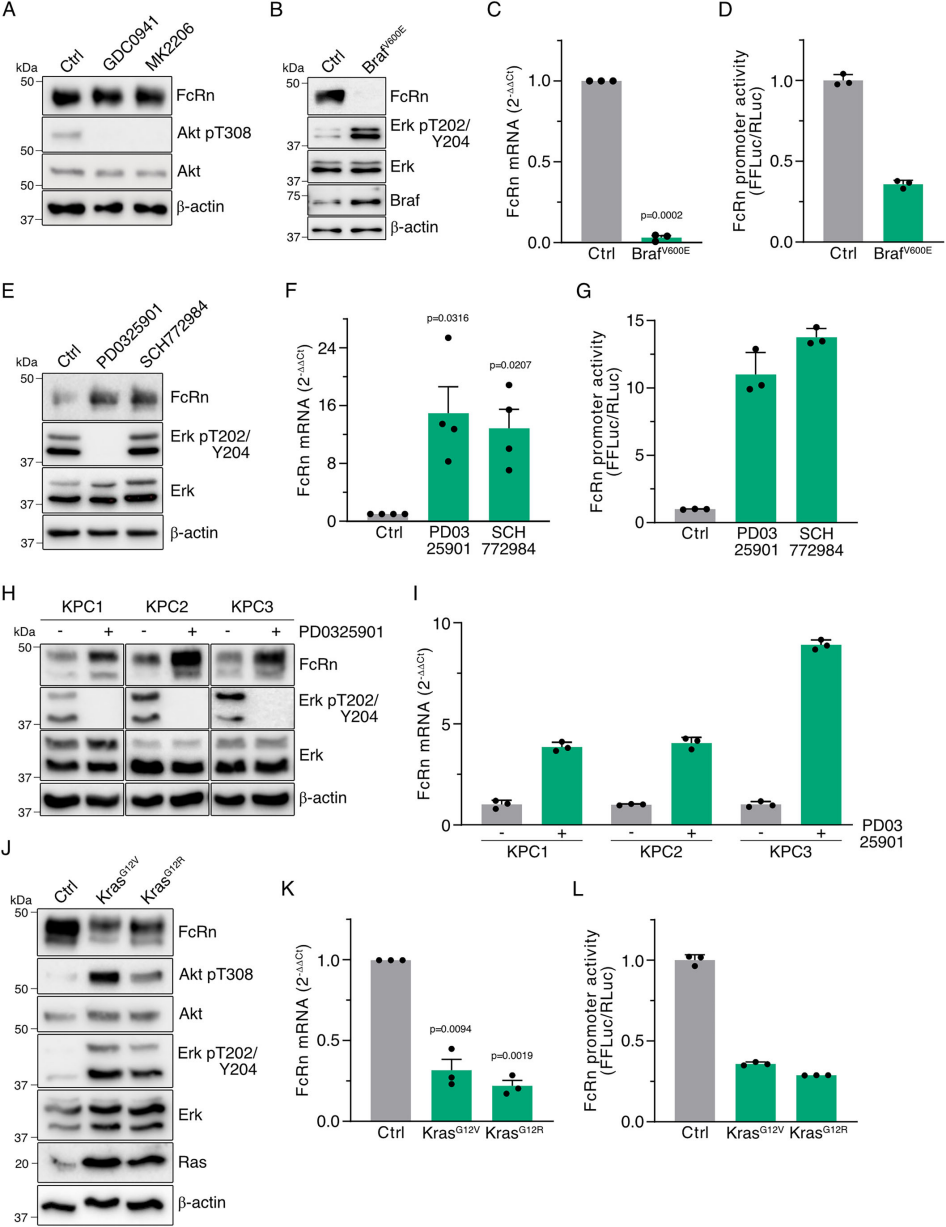
Ras represses FcRn expression through the MAPK signaling pathway. (**A**) FcRn protein abundance in MEFs after 16 h Hras^G12V^ induction and treatment with inhibitors against PI3-kinase (1 μM GDC0941) or Akt (2 μM MK2206), analyzed by immunoblotting. (**B**–**D**) FcRn regulation in MEFs after 16 h Braf^V600E^ induction; (**B**) FcRn protein abundance, analyzed by immunoblotting; (**C**) relative FcRn mRNA abundance, analyzed by RT-qPCR; (**D**) relative FcRn promoter activity, analyzed by dual-luciferase reporter assay. (**E**–**G**) FcRn regulation in MEFs after 16 h induction of Hras^G12V^ and treatment with inhibitors against Mek (2 μM PD0325901) or Erk (1 µM SCH772984); (**E**) FcRn protein abundance, analyzed by immunoblotting; (**F**) relative FcRn mRNA abundance, analyzed by RT-qPCR; (**G**) relative FcRn promoter activity, analyzed by dual-luciferase reporter assay. (**H**,** I**) FcRn regulation in pancreatic cancer KPC cells after 16 h treatment with Mek inhibitor (2 μM PD0325901); (**H**) FcRn protein abundance, analyzed by immunoblotting; (**I**) relative FcRn mRNA abundance, analyzed by RT-qPCR. (**J**–**L**) FcRn regulation in MEFs after 16 h Kras^G12V^ or Kras^G12R^ induction; (**J**) FcRn protein abundance, analyzed by immunoblotting; (**K**) relative FcRn mRNA abundance, analyzed by RT-qPCR; (**L**) relative FcRn promoter activity, analyzed by dual-luciferase reporter assay. All experiments were performed in 0.1% FBS. Data information: (**A**,** B**,** E**,** H**,** J**) Representative data from one out of n = 3 independent experiments. (**C**,** F**,** K**) Data are normalized replicate mean ± SEM (**C**, **K** *n* = 3; **F** *n* = 4 independent experiments). *p* values were calculated using a two-tailed unpaired *t*-test with Welch correction. (**D**,** G**,** L**) Data are mean ± SD (three technical replicates from one representative out of *n* = 2–3 independent experiments). (**I**) Data are mean ± SD (three technical replicates from one representative out of *n* = 2 independent experiments in three different cell lines). Source data are available online for this figure.

**Figure EV3:**
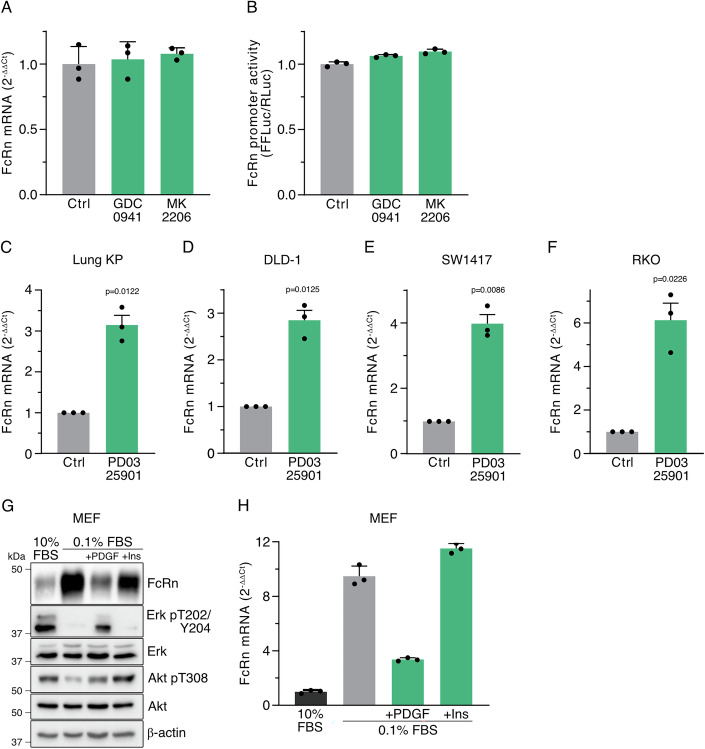
FcRn expression is regulated by Erk MAPK but not by PI3-kinase signaling. (**A**, **B**) FcRn regulation in MEFs after 16 h Hras^G12V^ induction and treatment with inhibitors against PI3-kinase (1 μM GDC0941) or Akt (2 μM MK2206); (**A**) relative FcRn mRNA abundance, analyzed by RT-qPCR; (**B**) relative FcRn promoter activity, analyzed by dual-luciferase reporter assay. (**C**–**F**) Relative FcRn mRNA abundance in cancer cells with indicated Kras–MAPK pathway mutations after 24 h treatment with Mek inhibitor (2 μM PD0325901), analyzed by RT-qPCR; (**C**) lung cancer KP, Kras^G12D^; (**D**) colorectal cancer DLD-1, Kras^G13D^; (**E**) colorectal cancer SW1417, Braf^V600E^; (**F**) colorectal cancer RKO, Braf^V600E^. (**G**,** H**) FcRn regulation in MEFs after 16 h in 10% FBS or 0.1% FBS + PDGF [2 nM] or insulin [4 μg/ml]; (**G**) FcRn protein abundance, analyzed by immunoblotting; (**H**) relative FcRn mRNA abundance, analyzed by RT-qPCR. If not otherwise indicated, experiments were performed in 0.1% FBS. Data information: (**A**,** B**,** H**) Data are mean ± SD (three technical replicates from one representative out of *n* = 2–3 independent experiments). (**C**–**F**) Data are normalized replicate mean ± SEM (*n* = 3 independent experiments). *p* values were calculated using a two-tailed unpaired *t*-test with Welch correction. For (**C**), controls are from Fig. 2F, for (**D**) from Fig. 2G. (**G**) Representative data from one out of *n* = 4 independent experiments. Source data are available online for this figure.

While our data linked constitutively active Ras to the transcriptional repression of FcRn, the responsible effector pathway downstream of Ras remained to be identified. One key Ras effector is the PI3-kinase pathway. To test whether PI3-kinase contributed to Ras-mediated FcRn repression, we treated Hras^G12V^ MEFs with pharmacological inhibitors of PI3-kinase or its downstream kinase Akt. Inhibition of either PI3-kinase or Akt did not affect the abundance of FcRn protein (Fig. 3A) and mRNA (Fig. EV3A) or FcRn promoter activity (Fig. EV3B). Thus, Ras does not regulate FcRn expression through PI3-kinase signaling.

Another critical Ras effector is the Raf–Mek–Erk MAPK signaling pathway. To determine the impact of MAPK signaling on FcRn expression, we enhanced MAPK activity by transducing MEFs with Braf^V600E^, an oncogenic, constitutively active Braf variant. Inducing Braf^V600E^ expression resulted in a strong reduction in FcRn protein abundance (Fig. 3B). Consistently, Braf^V600E^ expression reduced FcRn mRNA abundance (Fig. 3C) and promoter activity (Fig. 3D). Next, we inhibited MAPK signaling by treating Hras^G12V^ MEFs with pharmacological inhibitors against the downstream kinases, Mek and Erk. Inhibition of Mek or Erk led to a strong increase in the abundance of FcRn protein (Fig. 3E) and mRNA (Fig. 3F), as well as a corresponding increase in FcRn promoter activity (Fig. 3G). To confirm that MAPK signaling regulated FcRn in Ras-transformed cancer cells, we inhibited Mek in multiple KPC cell lines derived from murine pancreatic tumors driven by Kras^G12D^. Indeed, MAPK inhibition led to a substantial upregulation of FcRn, both at the protein and mRNA levels (Fig. 3H,I). Similarly, MAPK inhibition increased FcRn transcript levels in several other cancer cell lines harboring oncogenic mutations in Kras or Braf (Fig. EV3C–F). Together, these results demonstrate that constitutively active Ras suppresses FcRn expression through the MAPK signaling pathway.

Previous studies established PI3-kinase as essential for triggering macropinocytosis downstream of Ras (Palm et al, 2017). To further dissect the relationship between PI3-kinase-induced macropinocytosis and MAPK-dependent downregulation of FcRn, we generated MEFs expressing either Kras^G12V^ or the atypical variant Kras^G12R^. This mutation is found in ~20% of pancreatic cancer cases and, unlike canonical Kras mutants, is impaired in the activation of PI3-kinase, providing a genetic tool to uncouple Ras effector pathways (Fig. 3J) (Hobbs et al, 2020). Whereas Kras^G12V^ induction led to increased uptake of high molecular weight dextran, a selective macropinocytic cargo, Kras^G12R^ did not have an effect—consistent with impaired activation of PI3-kinase and macropinocytosis (Fig. EV1A,B). By contrast, Kras^G12R^ expression led to a marked decrease in FcRn protein and mRNA abundance as well as FcRn promoter activity, comparable to the effects observed for Kras^G12V^ (Fig. 3J–L). These findings further support the notion that Ras suppresses FcRn expression through MAPK signaling while enhancing macropinocytosis through PI3-kinase.

### Growth factor signaling suppresses FcRn expression in non-transformed cells

**Figure 4:**
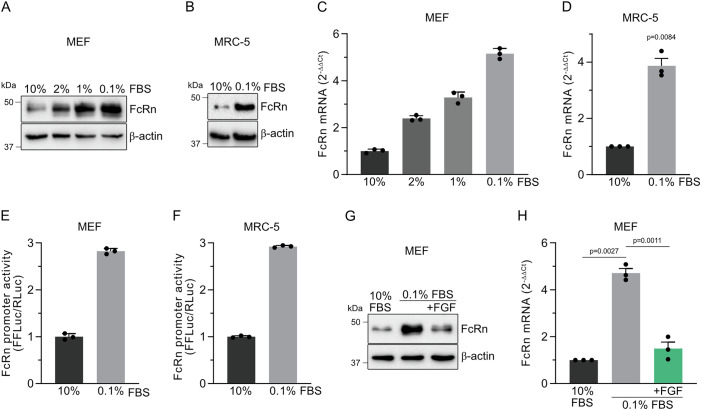
Growth factor stimulation represses FcRn in non-transformed cells. (**A**, **B**) FcRn protein abundance after 16 h in indicated FBS concentrations, analyzed by immunoblotting; (**A**) wild-type MEFs; (**B**) MRC-5 fibroblasts. (**C**, **D**) Relative FcRn mRNA abundance after 16 h in indicated FBS concentrations, analyzed by RT-qPCR; (**C**) wild-type MEFs; (**D**) MRC-5 fibroblasts. (**E**, **F**) Relative FcRn promoter activity after 16 h in 10 or 0.1% FBS, analyzed by dual-luciferase reporter assay; (**E**) wild-type MEFs; (**F**) MRC-5 fibroblasts. (**G**, **H**) FcRn regulation in wild-type MEFs after 16 h in 10% FBS or 0.1% FBS ± FGF [50 ng/ml]; (**G**) FcRn protein abundance, analyzed by immunoblotting; (**H**) relative FcRn mRNA abundance, analyzed by qPCR. Data information: (**A**,** B**,** G**) Representative data from one out of *n* = 3 independent experiments. (**C**) RT-qPCR data are mean ± SD (three technical replicates from one representative out of *n* = 3 independent experiments). (**D**,** H**) Data are normalized replicate mean ± SEM (*n* = 3 independent experiments). p values were calculated using a two-tailed unpaired *t*-test with Welch correction. (**E**,** F**) Data are mean ± SD (three technical replicates from one representative out of *n* = 3 independent experiments). Source data are available online for this figure.

Whereas oncogenic Ras mutants are constitutively active, physiological activation of Ras results from exogenous stimulation by growth factors. To investigate whether growth factor signaling can also regulate FcRn expression, we examined FcRn expression in wild-type MEFs cultured in media containing decreasing concentrations of FBS (10–0.1%). After 16 h, FcRn protein abundance progressively increased as FBS concentrations declined (Fig. 4A). Similarly, FBS deprivation resulted in a strong increase in FcRn abundance in MRC-5 fibroblasts (Fig. 4B). Consistently, subjecting MEFs and MRC-5 fibroblasts to FBS deprivation led to increased FcRn mRNA abundance (Fig. 4C,D) and promoter activity (Fig. 4E,F). To directly test whether this effect was mediated by the growth factors present in serum, we cultured MEFs in FBS-deprived medium supplemented with either fibroblast growth factor (FGF) or platelet-derived growth factor (PDGF). Addition of either FGF or PDGF was sufficient to suppress FcRn expression in serum-deprived cells, both at the levels of protein (Figs. 4G and EV3G) and mRNA (Figs. 4H and EV3H). In contrast, insulin—which predominantly activates PI3-kinase rather than MAPK signaling—did not abrogate the increased FcRn expression resulting from serum deprivation (Fig. EV3G,H). These results indicate that growth factor–MAPK signal transduction can suppress FcRn in non-transformed cells.

### FcRn antagonizes the nutritional utilization of albumin

**Figure 5:**
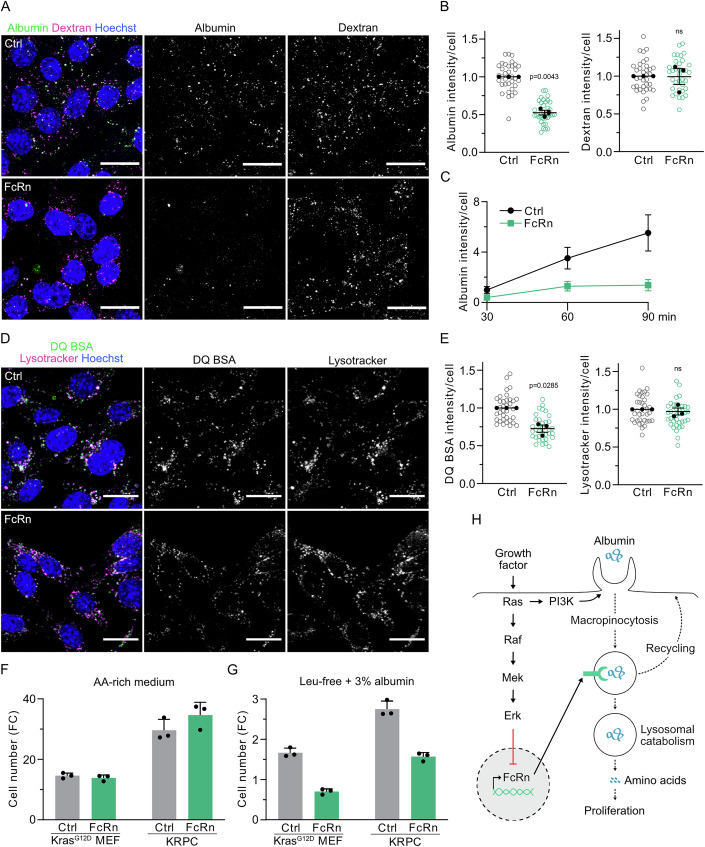
FcRn antagonizes albumin catabolism. (**A**) Intracellular levels of fluorescently labeled albumin and 10 kDa dextran in Kras^G12D^ MEFs with FcRn expression after 1 h uptake, analyzed by microscopy. Scale bars = 20 µm. (**B**) Quantification of albumin and dextran fluorescence of cells shown in (**A**). (**C**) Intracellular albumin accumulation in Kras^G12D^ MEFs with FcRn expression at the indicated time points. (**D**) DQ BSA degradation in Kras^G12D^ MEFs expressing FcRn after 5 h DQ BSA uptake, analyzed by microscopy. Lysosomes are labeled with lysotracker. Scale bars = 20 µm. (**E**) Quantification of DQ BSA and lysotracker fluorescence of cells shown in (**D**). (**F**, **G**) Fold change (FC) in cell number of Kras^G12D^ MEFs and murine pancreatic cancer KRPC cells; (**F**) 3 d in amino acid-rich medium; (**G**) 4 d in leucine-free medium + 3% albumin. (**H**) Model: Ras promotes the utilization of albumin as an amino acid source by suppressing FcRn through Raf–Mek–Erk MAPK signaling. The resulting decrease in recycling, together with Ras–PI3-kinase-mediated increase in macropinocytic albumin uptake, promotes lysosomal albumin degradation, creating an intracellular amino acid source that supports cancer cell growth in nutrient-poor environments. Data information: (**B**,** E**) Data are normalized replicate mean ± SEM (closed circles; *n* = 3 independent experiments) and fields of view (open circles; ≥10 per replicate). *p* values were calculated using a two-tailed unpaired *t*-test with Welch correction (ns not significant). (**C**) Data are mean ± SD (8 fields of view; one representative out of *n* = 3 independent experiments). (**F**,** G**) Data are mean ± SD (3 technical replicates; one representative out of *n* = 2–3 independent experiments in 2 independent cell lines). Source data are available online for this figure.

The above results raised the question of whether FcRn downregulation was required for the ability of Ras to promote the utilization of albumin as a nutrient source. To address this, we ectopically expressed FcRn and its binding partner, B2m, in Kras^G12D^ MEFs and examined the effect on the internalization of fluorescently labeled albumin. Ectopic FcRn expression led to a significant decrease in intracellular albumin accumulation (Fig. 5A,B). This difference was already detectable after 30 min of albumin uptake and became more pronounced over time: whereas Kras^G12D^ MEFs progressively accumulated albumin, intracellular albumin levels remained low in FcRn-expressing cells (Fig. 5C). Of note, ectopic FcRn expression did not affect the uptake of dextran, indicating that decreased intracellular albumin accumulation was not the result of a general decrease in endocytosis (Fig. 5A,B). To determine whether FcRn prevents lysosomal degradation of albumin, we examined the impact of FcRn on DQ BSA degradation. Ectopic FcRn expression significantly decreased DQ BSA fluorescence dequenching, without affecting lysosomal abundance or acidification (Fig. 5D,E). Thus, FcRn expression hinders Kras-transformed cells from accumulating and lysosomally degrading albumin.

**Figure EV4:**
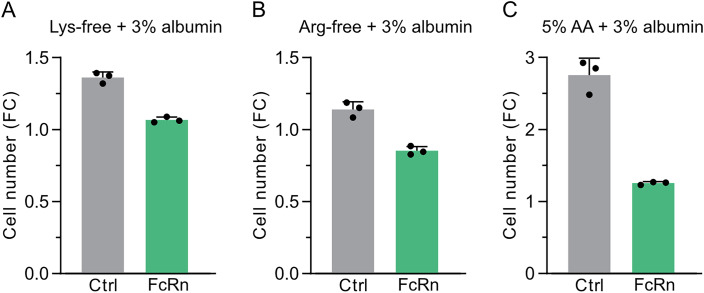
FcRn suppresses the proliferation of cells that depend on albumin as a nutrient. (**A**–**C**) Fold change (FC) in cell number of Kras^G12D^ MEFs after 4 days in amino acid-deficient media supplemented with 3% albumin; (**A**) lysine-free; (**B**) arginine-free; (**C**) all amino acids at 5% of standard medium (DMEM/F-12). Data information: (**A**–**C**) Data are mean ± SD (three technical replicates from one representative out of *n* = 2–3 independent experiments). Source data are available online for this figure.

Finally, we investigated whether FcRn could counteract the ability of Kras-transformed cells to utilize albumin as an amino acid source. To this end, we ectopically expressed FcRn in two Kras-transformed cell lines that grow robustly using albumin as a nutrient: Kras^G12D^ MEFs and pancreatic cancer KRPC cells (Palm et al, 2015). Cells were cultured in standard amino acid-rich medium or in leucine-deficient medium supplemented with physiological albumin levels (3%). In standard medium, FcRn expression did not affect cell proliferation, consistent with the plentiful supply of free amino acids (Fig. 5F). In leucine-free medium supplemented with 3% albumin, however, the proliferation of Kras^G12D^ MEFs and KRPC cells was markedly suppressed by FcRn expression (Fig. 5G). As albumin serves as a source of all proteinogenic amino acids, we further tested whether FcRn could suppress growth in media lacking other essential amino acids (lysine or arginine) or containing overall low amino acid levels (5% of complete medium). FcRn expression reduced the viability/proliferation of Kras^G12D^ MEFs across the different albumin-supplemented, amino acid-deficient conditions (Fig. EV4). These results suggest that Ras-mediated downregulation of FcRn is critical to support the adaptation of transformed cells to nutrient environments in which they depend on albumin as an amino acid source.

The present study uncovers a novel function of Ras in regulating the albumin recycling receptor, FcRn. We demonstrate that cancer-associated Ras mutants suppress FcRn expression through downstream MAPK signaling, promoting the growth of cancer cells that exploit uptake and lysosomal degradation of albumin to acquire amino acids (Fig. 5H). In addition to FcRn downregulation, oncogenic Ras enhances macropinocytosis through activation of PI3-kinase (Amyere et al, 2000; Palm et al, 2017). This suggests that the efficient degradation of albumin characteristic of Ras-transformed cells results from the concerted increase in cellular uptake and suppression of endosomal recycling. Notably, albumin accounts for more than 50% of total plasma protein (Anderson and Anderson, 2002). FcRn also recycles immunoglobulin G, which constitutes more than 10% of plasma protein and could potentially serve as an additional nutrient source. Whereas free amino acid levels are often decreased or fluctuating in tumors, plasma proteins can accumulate due to leaky vasculature and poor lymphatic drainage, a phenomenon referred to as the enhanced permeability and retention (EPR) effect (Fang et al, 2011). By facilitating access to the abundant amino acid stores of extracellular proteins, FcRn suppression may thus provide a selective advantage for Ras-transformed cells.

Pharmacological inhibitors of Ras, MAPK and PI3-kinase exert anti-cancer effects in part by targeting the metabolic dysregulation driven by these oncogenic signaling pathways (Ebright et al, 2025; Lien et al, 2017). Evidence suggests that inhibiting different Ras effector pathways has distinct metabolic consequences: MAPK inhibition restores FcRn expression, whereas PI3-kinase inhibition blocks macropinocytosis. Inhibiting PI3-kinase signaling further downstream via Akt or mTORC1 can even have the opposite effect, stimulating lysosomal nutrient generation and promoting the growth of Ras-transformed cells that feed on extracellular proteins (Palm et al, 2017; Ratto et al, 2022). Thus, the impact of these different inhibitors on endo-lysosomal nutrient acquisition is likely influenced by the relevance of albumin compared to other extracellular proteins as nutrient sources in tumors. Our results further raise the possibility that FcRn suppression could affect the response of Ras-driven cancers to protein-based therapeutics. Albumin-conjugated paclitaxel (Nab-paclitaxel) shows improved efficacy in pancreatic cancer and several other cancers (Sand et al, 2014; Springfeld et al, 2023; Von Hoff et al, 2013), and FcRn depletion increases the sensitivity to albumin-conjugated doxorubicin in a mouse pancreatic cancer model (Liu et al, 2019). These observations raise the possibility that FcRn suppression could modulate the sensitivity of Ras-driven cancers to albumin-conjugated drugs. Since FcRn-mediated recycling is a critical determinant of IgG half-life in circulation (Pyzik et al, 2019; Roopenian et al, 2003), Ras-driven suppression of FcRn could have further implications for the use of antibody-based therapeutics.

The contribution of albumin degradation in tumors to whole-body albumin turnover remains poorly defined. However, studies in rodent transplantation models have reported substantial albumin degradation in tumors (Stehle et al, 1997), as well as hypoalbuminemia—low plasma albumin concentrations—resulting from FcRn depletion in cancer cells (Swiercz et al, 2017). Ras-driven cancers and hypoalbuminemia are associated with cancer cachexia, a wasting syndrome characterized by severe loss of skeletal muscle and adipose tissue (Ni and Zhang, 2020). Conceivably, FcRn downregulation by oncogenic Ras signaling contributes to hypoalbuminemia and cachexia by increasing albumin degradation in tumors. Because our results suggest that oncogenic Ras co-opts the normal regulation of FcRn by growth factor signaling, regulated FcRn expression could have implications for nutrient acquisition, plasma protein homeostasis and therapeutic responses in a range of physiological contexts.

## Methods

**Table:** Reagents and tools table

Reagent/resource	Reference or source	Identifier or catalog number
**Experimental models**		
EPP2	(Groessl et al, 2025)	
HEK 293 T	ATCC	CRL-3216
KPC	(Hingorani et al, 2005)	
KRPC	(Palm et al, 2015)	
MRC-5	ATCC	CCL-171
SV40 large T-immortalized MEFs *(Kras wt* and *G12D)*	(Palm et al, 2015)	
Lung KP	(Jackson et al, 2005)	
DLD-1	ATCC	CCL-221
SW1417	ATCC	CCL-238
RKO	ATCC	CRL-2577
**Recombinant DNA**		
psPAX2	Addgene	12260
pCMV-VSV-G	Addgene	8454
Gag-Pol	Addgene	14887
pBabe-HrasG12V (human)	(Palm et al, 2015)	
pTURN-HrasG12V (human)	(Palm et al, 2015)	
pTURN-KrasG12V (mouse)	(Palm et al, 2015)	
pTURN-BrafV600E (human)	This study	
pTURN-KrasG12R (mouse)	This study	
pBabe-FcRn-P2A-B2m (human)	This study	
**Antibodies**		
Akt pT308	Cell Signaling Technology	9275
Akt(pan)	Cell Signaling Technology	2920
β-actin	Sigma-Aldrich	A5441
Braf	Proteintech	20899-1-AP
Erk1/2	Cell Signaling Technology	9107
Erk1/2 pT202/Y204	Cell Signaling Technology	9101
FcRn (human)	Novus Biologicals	NBP1-89128
FcRn (mouse)	R&D Systems	AF6775
Ras (pan)	Cell Signaling Technology	3965
HRP anti-goat	Life Technologies	31402
HRP anti-mouse	Cytiva	NA931
HRP anti-rabbit	Cytiva	NA9340
**Oligonucleotides and other sequence-based reagents**		
qPCR primers	This study	
**Chemicals, enzymes, and other reagents**		
Alexa Fluor 647 BSA	Life Technologies	A34785
DQ Green BSA	Life Technologies	D12050
FITC Dextran 10 kDa	Life Technologies	D1821
Hoechst 33342	Life Technologies	H1399
Lysotracker Red	Life Technologies	L7528
Oregon Green 488 Dextran 70 kDa	Life Technologies	D7172
Actinomycin D	SERVA Electrophoresis	10710
GDC0941	Cayman Chemical	957054-30-7
MK2206	Cayman Chemical	1032350-13-2
PD0325901	Cayman Chemical	391210-10-9
SCH772984	Selleck Chemicals	S7101
BI-2865	MedChemExpress	HY-153724
Sotorasib (AMG510)	Selleck Chemicals	S8830
Halt Protease and Phosphatase Inhibitor Cocktail	Thermo Fisher Scientific	78444
Mouse FGF-basic (FGF-2/bFGF) Recombinant Protein	Thermo Fisher Scientific	450-33
Mouse PDGF-BB Recombinant Protein	Life Technologies	PMG0044
Insulin	Life Technologies	12585014
BamHI-HF	NEB	R3136
BbsI-HF	NEB	R3539
FastDigest Esp3I	Thermo Fisher Scientific	FD0454
Q5 High-Fidelity DNA Polymerase	NEB	M0491L
Trizol	Invitrogen	15596026
Chloroform	Carl Roth	7331
Isopropanol	Serva	39559
Ethanol	Th. Geyer	11832330
Random hexamers	Invitrogen	N8080127
SuperScript III Reverse Transcriptase	Invitrogen	18080093
SYBR green master mix	Life Technologies	4309155
T4 Polynucleotide Kinase	NEB	M0201
T7 DNA Ligase	NEB	M0318
Taq DNA polymerase	Thermo Fisher Scientific	EP0401
Dual-Luciferase Reporter Assay System	Promega	E1910
Q5 Site-Directed Mutagenesis Kit	NEB	E0554S
NEBuilder HiFi DNA Assembly Cloning Kit	NEB	E2621L
Pierce BCA Protein Assay Kit	Thermo Scientific	23227
DMEM	Gibco	41965-039
DMEM/F-12	Gibco	11320-074
Amino acid-free, glucose-free DMEM/F-12	US Biological	D9807-11
Dialyzed FBS	Gibco	26400-044
FBS	Gibco	10270-106
Glutamine	Gibco	25030-024
Sodium bicarbonate	Sigma-Aldrich	S5761
Glucose	Sigma-Aldrich	G7021
Bovine Serum Albumin	Sigma-Aldrich	A1470
Amino acids	Sigma-Aldrich	
Polybrene	Tocris	7711
Polyethylenimine (PEI, MW 250000)	Polysciences	24314
Geneticin (G418)	Gibco	10131-035
Blasticidin	Santa Cruz Biotechnology	sc-495389
Hygromycin	Gibco	10687-010
Doxycycline	Sigma-Aldrich	J67043
**Software**		
Image Lab (v6.0.0.25)	Bio-Rad	
GraphPad Prism (v10.1.0)	Prism	
NEBbuilder Assembly Tool	nebuilder.neb.com	
Fiji	(Schindelin et al, 2012)	
**Other**		
CASY Cell Counter and Analyzer	OMNI Life Sciences	
ChemiDoc Imaging System	Bio-Rad	
GelDoc Go Imaging System	Bio-Rad	
Infinite 200 Pro plate reader	Tecan	
Synergy H1 plate reader	BioTek	
LightCycler 480	Roche	
LSM 900 confocal microscope	Zeiss	
NanoDrop One	Thermo Fisher Scientific	
SP8 confocal microscope	Leica	

### Cell culture

Fibroblast and cancer cell lines were cultured in DMEM/F-12 supplemented with 2 mM glutamine and, if not otherwise indicated, 10% FBS. HEK 293T cells were cultured in DMEM supplemented with 2 mM glutamine and 10% FBS. Cells were cultured at 37 °C and 5% CO_2_, authenticated by SNP typing (Multiplexion) and routinely tested for mycoplasma contamination (MycoAlert Mycoplasma Detection, Lonza).

### Cell proliferation assays

For proliferation experiments, cells were seeded in complete medium, allowed to attach for at least 5 h, rinsed with PBS and cultured in the different media conditions for the indicated times. Amino acid-deficient media and amino acid-replete media were prepared from reconstituted amino acid-free, glucose-free DMEM/F-12. Media were supplemented with sodium bicarbonate, glucose and 10% dialyzed FBS. Amino acids (Sigma-Aldrich) were supplemented at DMEM/F-12 standard concentrations (amino acid-rich) or at 5% of DMEM/F-12 concentrations; arginine, leucine or lysine were omitted as indicated. BSA was supplemented at physiological concentrations (3% w/v). Cells were counted using a CASY Cell Counter and Analyzer.

### Viral transduction and cloning

Lentivirus was produced by co-transfecting HEK 293T cells with the lentiviral plasmid of interest, psPAX2 and pCMV-VSV-G. Retrovirus was produced by co-transfecting HEK 293T cells with the retroviral plasmid of interest, Gag-Pol and pCMV-VSV-G. Polyethylenimine was used as a transfection reagent. 48 to 72 h after transfection, virus-containing supernatants were collected, filtered through a 0.45 µm PES membrane and added to target cells with the addition of 10 µg/ml polybrene.

In MEFs, constitutively active oncogenes were expressed under the control of a doxycycline-inducible promoter from a modified version of the retroviral vector pTRE-Tight (pTURN) as previously described (Palm et al, 2015). pTURN-Kras^G12R^ was derived from pTURN-Kras^G12V^ using site-directed mutagenesis. pTURN- Braf^V600E^ was subcloned from pBabe-Puro-Braf-V600E (Addgene 15269). MEFs were stably transduced with the indicated oncogenes in pTURN, and gene expression was induced by the addition of doxycycline (Hras, 0.1 µg/ml; Kras, Braf, 1 µg/mL) to culture media for 16 h. MRC-5 fibroblasts were transduced with Hras^G12V^ in a stable expression vector (pBabe).

The coding sequence of human FcRn was amplified from a cDNA clone plasmid (Origene SC117595), and the coding sequence of B2m was amplified from a human cDNA library. Both sequences were subcloned into pBabe (Addgene 21836) to generate a bicistronic construct separated by a P2A self-cleaving site. All transduced cells were selected with the appropriate antibiotic prior to downstream experiments.

### Western blotting

Cells were lysed in ice-cold lysis buffer (50 mM HEPES pH 7.4, 40 mM NaCl_2_, 2 mM EDTA, 1 mM sodium orthovanadate, 50 mM sodium fluoride, 10 mM sodium pyrophosphate, 10 mM sodium glycerophosphate, 1% Triton X-100, 1x Halt protease and phosphatase inhibitor cocktails) for 15 min, and the soluble lysate fraction was isolated by centrifugation at 18,000 × *g* for 5 min at 4 °C. Protein concentrations were determined using the Pierce BCA Protein Assay (Thermo Fisher Scientific). Equal amounts of protein were loaded on SDS gels, and gel electrophoresis was performed following standard protocols. Proteins were transferred to nitrocellulose membranes and incubated with primary antibodies. After incubation with HRP-conjugated secondary antibodies, membranes were developed using the ECL substrate Clarity Max Western (Bio-Rad). The chemiluminescence signal was detected using a ChemiDoc Touch imaging system (Bio-Rad). To analyze multiple antibodies in different membranes, sample preparation, electrophoresis, and immunoblotting were performed in parallel and under identical conditions.

### RT-qPCR

Total RNA was extracted with Trizol (Invitrogen) according to the manufacturer’s instructions and quantified using a NanoDrop One. cDNA was synthesized from 1 to 2 µg RNA with SuperScript III Reverse Transcriptase and random hexamers from Invitrogen. qPCR was performed using SYBR Green Master Mix. A minimum of three replicate samples were run, each in duplicate, and averaged. Relative gene expression was calculated using the delta-delta Ct method (2^−ΔΔCt^). The geometric mean of two reference genes was used to normalize gene expression. For mouse cell lines, *Tbp* and *Gapdh* were used as reference genes; in human cell lines, *PPIA* and *GAPDH* were used. qPCR primers are listed in Table 1 below.

**Table 1:** RT-qPCR primers.

Gene	Species	Forward primer (5’ → 3’)	Reverse primer (5’ → 3’)
FCGRT	human	TTCCTGCTATTCTCCTGCCCGCAC	AAGCACGGAAAAGCCAGGGCTG
Fcgrt	mouse	CCTGTGGTTGGAATCGTTCT	GTTCCCACCAGGCAACA
GAPDH	human	AGATCCCTCCAAAATCAAGTGG	GGCAGAGATGATGACCCTTTT
Gapdh	mouse	TCACCACCATGGAGAAGGC	GCTAAGCAGTTGGTGGTGCA
PPIA	human	TCTTTCACTTTGCCAAACACC	CATCCTAAAGCATACGGGTCC
Tbp	mouse	CCTTGTACCCTTCACCAATGAC	ACAGCCAAGATTCACGGTAGA

mRNA stability was evaluated by blocking transcription with actinomycin D [5 µg/mL] for different time intervals (0–8 h) before total RNA extraction. Actinomycin D treatment was adapted from previously described protocols (Qiu et al, 2016). RNA extraction, cDNA synthesis and RT-qPCR were performed as previously described. Fold changes obtained from the 2^−ΔΔCt^ analysis were used to generate nonlinear fit one-phase decay curves with GraphPad Prism 10.

### Luciferase reporter assay

Firefly luciferase promoter activity reporter vectors were constructed by replacing the EF1A promoter of the Lenti-luciferase-P2A-Neo vector (Addgene 105621) with the promoter regions of human (−441/+272) or mouse (−2289/+78) FcRn. Base pair positions are indicated relative to the transcriptional start site. A promoterless control luciferase was generated by removing the EF1A promoter. Cells were stably transduced with firefly luciferase reporters and pLenti.PGK.blast-Renilla_Luciferase (Addgene 74444), which constitutively expresses Renilla luciferase. Dual-luciferase activity was measured using a Dual-Luciferase Reporter Assay (Promega E1910). Firefly luciferase-produced luminescence was normalized to Renilla luciferase-produced luminescence to account for overall translation in each experimental group. Resulting values were normalized to the experimental control group.

### Fluorescence microscopy

For bovine serum albumin (BSA) and dextran uptake experiments, cells were seeded on µ-Slide 8-well coverslips (IBIDI) and left to attach overnight. Prior to imaging, cells were incubated with 0.1 mg/mL Oregon Green 488 70 kDa dextran, FITC 10 kDa dextran or Alexa Fluor 647 BSA for times as indicated in figure legends. Cells were then washed with PBS, fixed with 4% paraformaldehyde for 15 min, and stained with 10 µg/mL Hoechst (all steps in PBS). Imaging was performed with a Leica SP8 confocal microscope using a 63x 1.40 oil objective.

Lysosomal BSA degradation was evaluated through live-imaging of DQ BSA fluorescence dequenching. Cells were seeded on µ-Slide 8-Well coverslips (IBIDI) and left to attach overnight. Prior to imaging, cells were incubated with 0.5 mg/mL DQ Green BSA for 5 h. Thirty minutes before imaging, the medium was supplemented with 10 nM Lysotracker Red and 10 µg/mL Hoechst. Live-cell imaging was performed in a humidified chamber at 37 °C and 5% CO_2_ with a Zeiss LSM 900 confocal microscope using a 40x or 63x 1.40 oil objective. Images were taken in randomly chosen fields of view across the entirety of the sample (>10 cells/field of view in all experiments)

Z‑stack images were projected using maximum intensity projection, and fluorescence per cell was measured using the Analyze Particles function of Fiji. Mean fluorescence/cell was calculated by dividing the integrated signal density of each fluorescent probe by the number of nuclei.

### Tumor expression analysis

Gene expression data from non-small cell lung cancer (NSCLC) and colorectal cancer (CRC) patients before and 8 days after treatment with the Kras^G12C^-selective inhibitor adagrasib were obtained from a previously published dataset (Klomp et al, 2024; data ref: Klomp et al, 2024). Normalized counts per million (CPM) were log2-transformed and *p* values were calculated using a two-tailed paired Student’s *t*-test. Analyses were performed using GraphPad Prism 10.

## Supplementary information

Peer Review File

Source data Fig. 1

Source data Fig. 2

Source data Fig. 3

Source data Fig. 4

Source data Fig. 5

EV Figures Source Data

Expanded View Figures

## Data Availability

This study includes no data deposited in external repositories. The source data of this paper are collected in the following database record: biostudies:S-SCDT-10_1038-S44319-026-00787-4.
