# Supplementary figures and images for: Ras promotes macropinocytic nutrient uptake by suppressing the albumin recycling receptor FcRn
Source: EMBO Rep. 2026 Apr 29;27(12):3214–30. doi: 10.1038/s44319-026-00787-4 (PMC13303908; doi:10.1038/s44319-026-00787-4)

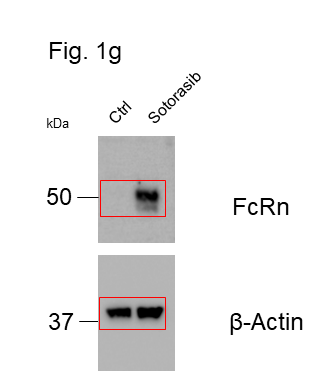

Supplement: Supplementary file 2 — Source data Fig. 1 [file 44319_2026_787_MOESM2_ESM.zip › 1G/Fig. 1g.tif]

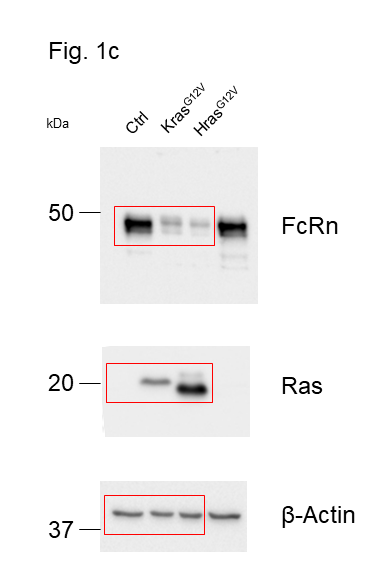

Supplement: Supplementary file 2 — Source data Fig. 1 [file 44319_2026_787_MOESM2_ESM.zip › 1C/Fig. 1c.tif]

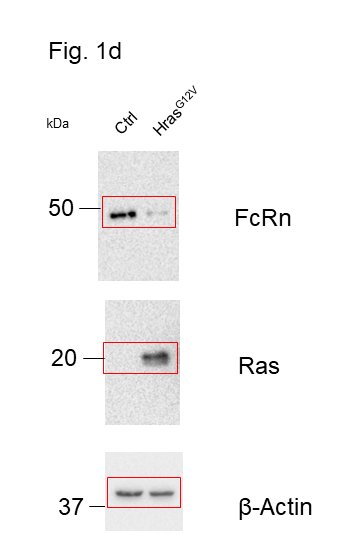

Supplement: Supplementary file 2 — Source data Fig. 1 [file 44319_2026_787_MOESM2_ESM.zip › 1D/Fig. 1d.TIF]

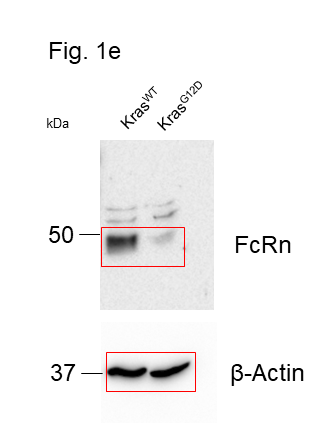

Supplement: Supplementary file 2 — Source data Fig. 1 [file 44319_2026_787_MOESM2_ESM.zip › 1E/Fig. 1e.tif]

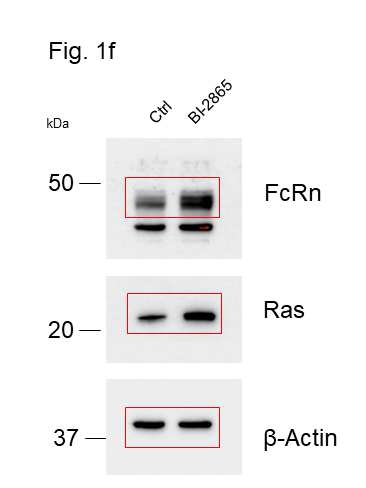

Supplement: Supplementary file 2 — Source data Fig. 1 [file 44319_2026_787_MOESM2_ESM.zip › 1F/Fig. 1f.tif]

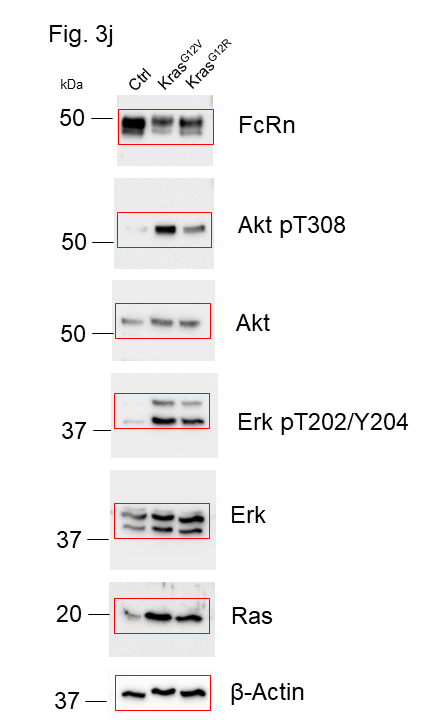

Supplement: Supplementary file 4 — Source data Fig. 3 [file 44319_2026_787_MOESM4_ESM.zip › 3J/Fig. 3j.tif]

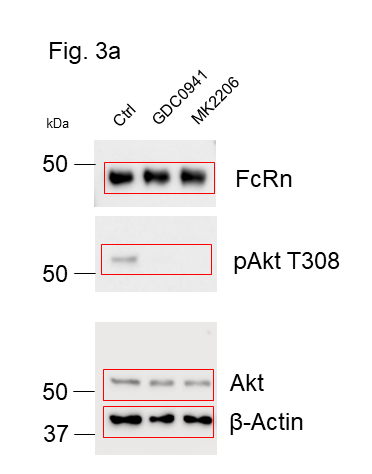

Supplement: Supplementary file 4 — Source data Fig. 3 [file 44319_2026_787_MOESM4_ESM.zip › 3A/Fig. 3a.tif]

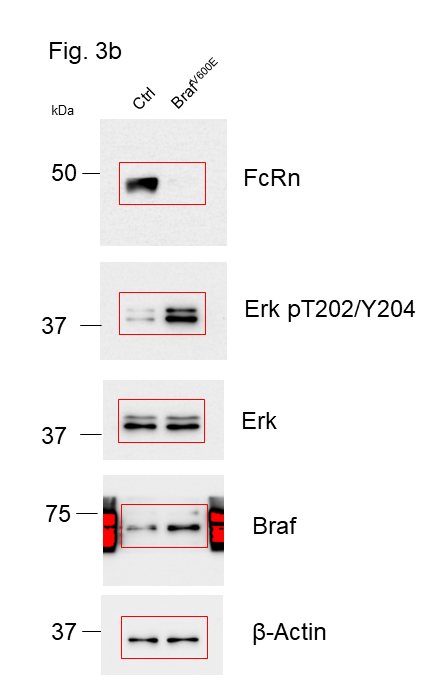

Supplement: Supplementary file 4 — Source data Fig. 3 [file 44319_2026_787_MOESM4_ESM.zip › 3B/Fig. 3b.tif]

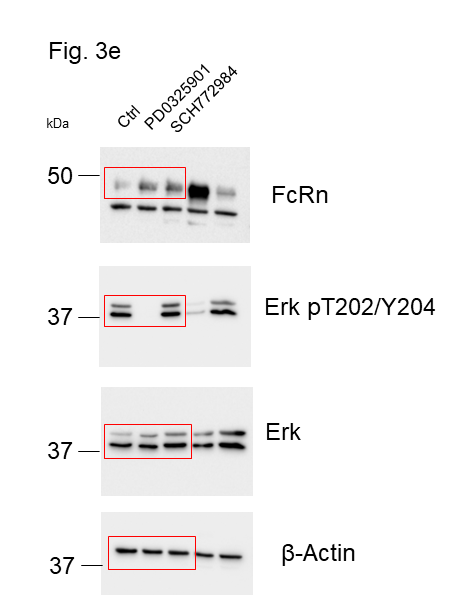

Supplement: Supplementary file 4 — Source data Fig. 3 [file 44319_2026_787_MOESM4_ESM.zip › 3E/Fig. 3e.tif]

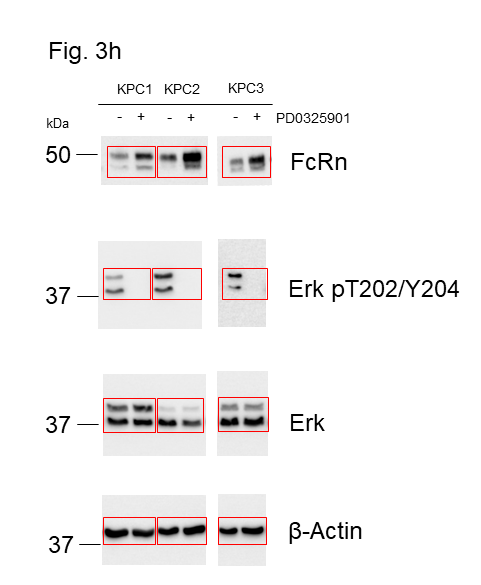

Supplement: Supplementary file 4 — Source data Fig. 3 [file 44319_2026_787_MOESM4_ESM.zip › 3H/Fig. 3h.tif]

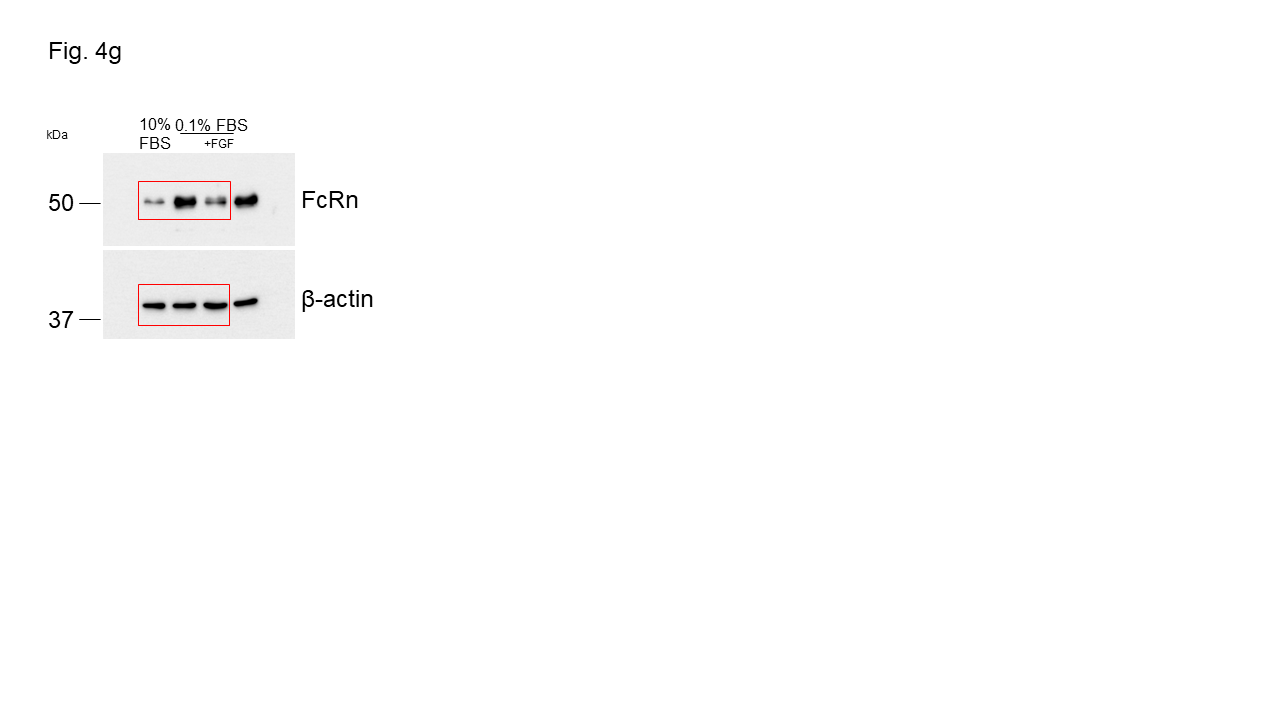

Supplement: Supplementary file 5 — Source data Fig. 4 [file 44319_2026_787_MOESM5_ESM.zip › 4G/Fig. 4g.tif]

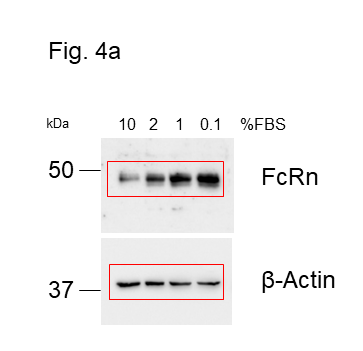

Supplement: Supplementary file 5 — Source data Fig. 4 [file 44319_2026_787_MOESM5_ESM.zip › 4A/Fig. 4a.tif]

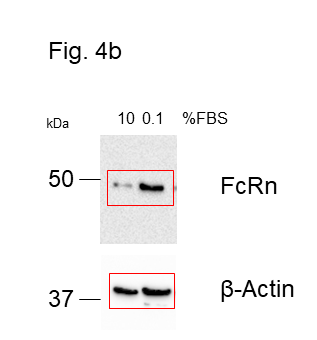

Supplement: Supplementary file 5 — Source data Fig. 4 [file 44319_2026_787_MOESM5_ESM.zip › 4B/Fig. 4b.tif]

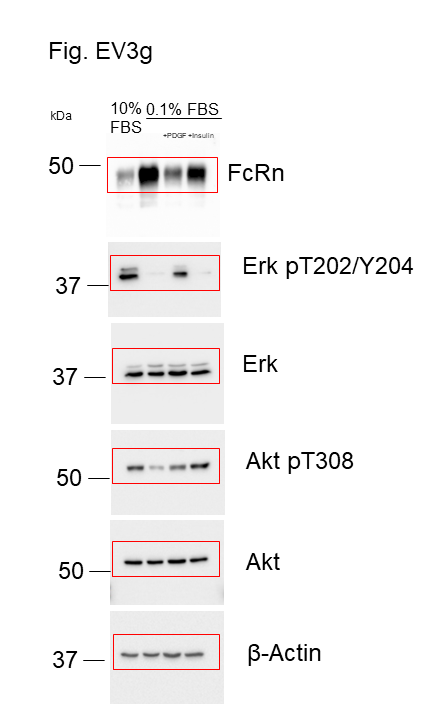

Supplement: Supplementary file 7 — EV Figures Source Data [file 44319_2026_787_MOESM7_ESM.zip › Fig. EV3/EV3G/Fig. EV3g.tif]
