# Supplementary material for: Ras promotes macropinocytic nutrient uptake by suppressing the albumin recycling receptor FcRn
Source: EMBO Rep. 2026 Apr 29;27(12):3214–30. doi: 10.1038/s44319-026-00787-4 (PMC13303908; doi:10.1038/s44319-026-00787-4)
Supplement: Supplementary file 8 — Expanded View Figures [file 44319_2026_787_MOESM8_ESM.pdf]

Expanded View Figures

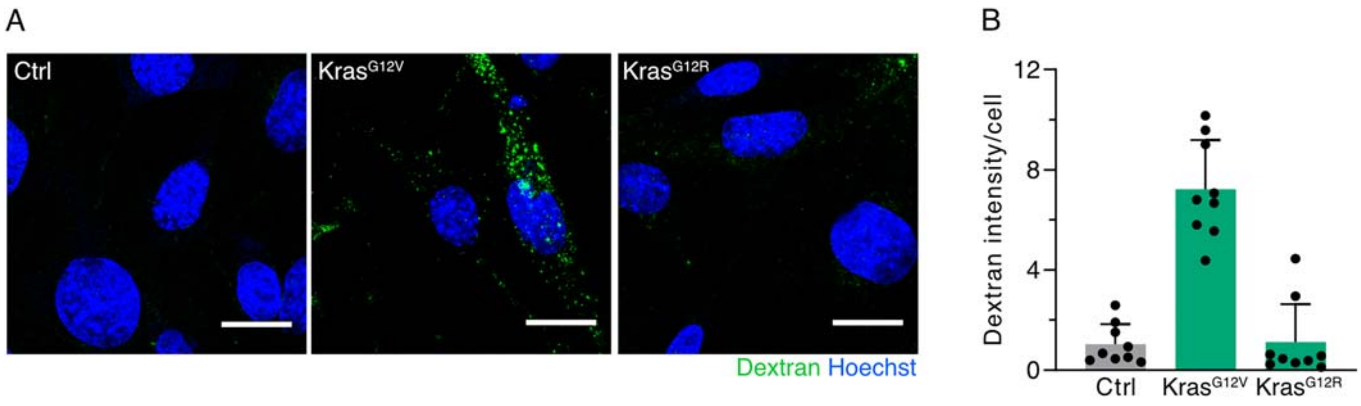

Figure EV1. Macropinocytosis induction by Kras^{G12V} but not by atypical Kras^{G12R}.

(**A**) Intracellular levels of 70 kDa dextran in MEFs after 16 h induction of Kras^{G12V} or Kras^{G12R} and 1 h dextran uptake. Scale bars = 20 µm. (**B**) Quantification of dextran fluorescence of cells shown in A. Experiments were performed in 0.1% FBS. Data information: (**B**) Data are mean ± SD (*n* = 9 fields of view from one representative out of *n* = 3 independent experiments). Source data are available online for this figure.

 © The Author(s)

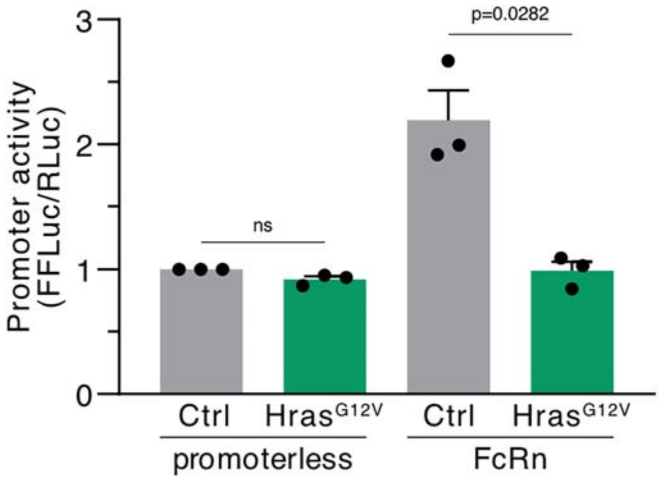

Figure EV2. The FcRn promoter is regulated by Ras signaling.

Relative luciferase expression in MEFs after 16 h induction of Hras^{G12V}, analyzed with dual-luciferase reporter assay (Firefly luciferase: FFLuc; Renilla luciferase: RLuc). Firefly luciferase was stably expressed in a basal or FcRn-regulated manner by transducing cells with Lenti-luciferase-P2A-Neo without promoter (promoterless) or under control of the FcRn promoter, followed by G418 (Neo) selection. Experiments were performed in 0.1% FBS. Data information: Data are normalized replicate mean ± SEM ($n = 3$ independent experiments with 3 technical replicates). p values were calculated using a two-tailed unpaired t-test with Welch correction (ns not significant). Source data are available online for this figure.

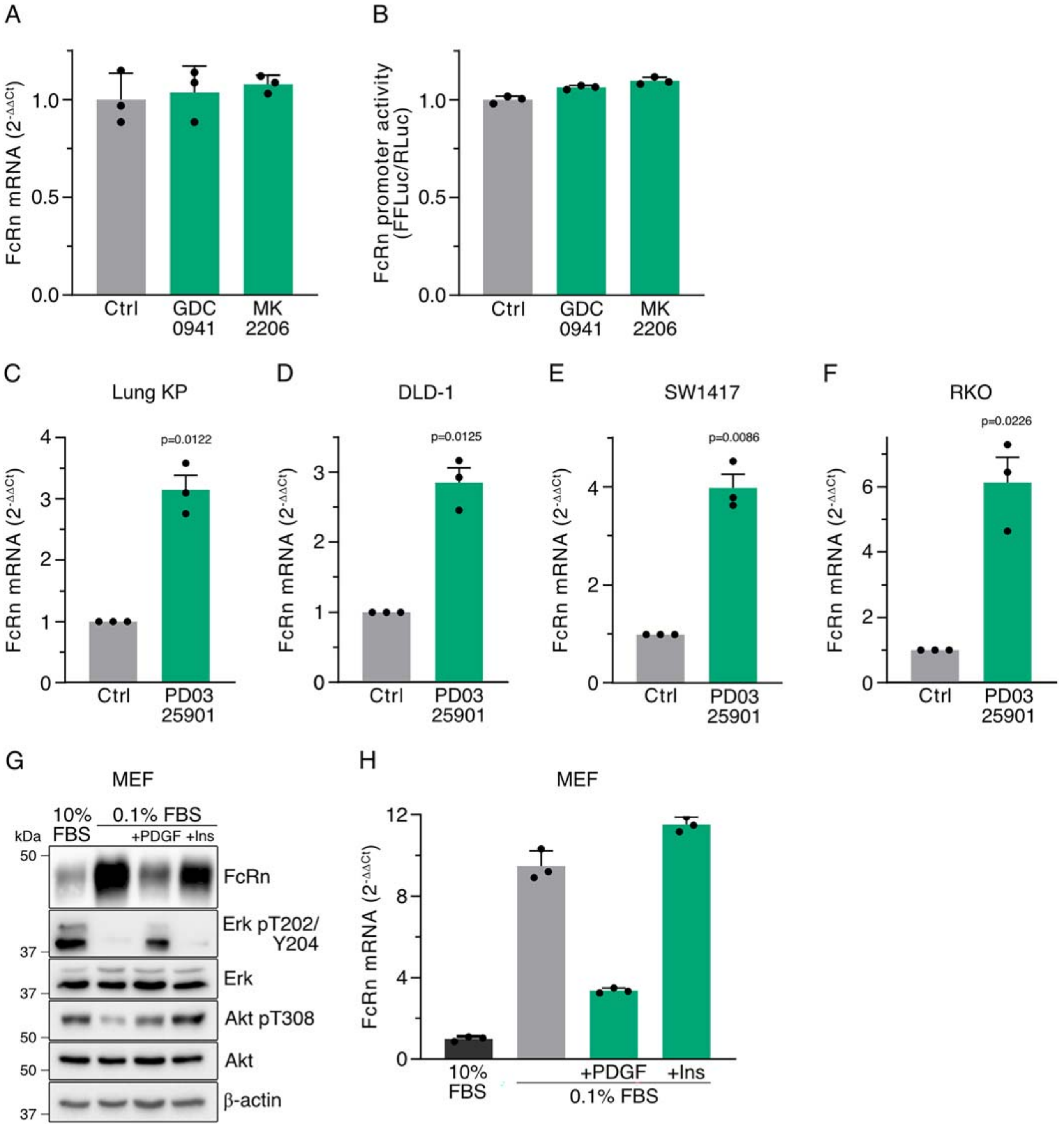

Figure EV3. FcRn expression is regulated by Erk MAPK but not by PI3-kinase signaling.

(**A**, **B**) FcRn regulation in MEFs after 16 h $Hras^{G12V}$ induction and treatment with inhibitors against PI3-kinase (1 µM GDC0941) or Akt (2 µM MK2206); (**A**) relative FcRn mRNA abundance, analyzed by RT-qPCR; (**B**) relative FcRn promoter activity, analyzed by dual-luciferase reporter assay. (**C–F**) Relative FcRn mRNA abundance in cancer cells with indicated Kras–MAPK pathway mutations after 24 h treatment with Mek inhibitor (2 µM PD0325901), analyzed by RT-qPCR; (**C**) lung cancer KP, $Kras^{G12D}$; (**D**) colorectal cancer DLD-1, $Kras^{G13D}$; (**E**) colorectal cancer SW1417, $Braf^{V600E}$; (**F**) colorectal cancer RKO, $Braf^{V600E}$. (**G**, **H**) FcRn regulation in MEFs after 16 h in 10% FBS or 0.1% FBS + PDGF [2 nM] or insulin [4 µg/ml]; (**G**) FcRn protein abundance, analyzed by immunoblotting; (**H**) relative FcRn mRNA abundance, analyzed by RT-qPCR. If not otherwise indicated, experiments were performed in 0.1% FBS. Data information: (**A**, **B**, **H**) Data are mean ± SD (three technical replicates from one representative out of $n = 2$–3 independent experiments). (**C–F**) Data are normalized replicate mean ± SEM ($n = 3$ independent experiments). p values were calculated using a two-tailed unpaired t-test with Welch correction. For (**C**), controls are from Fig. 2F, for (**D**) from Fig. 2G. (**G**) Representative data from one out of $n = 4$ independent experiments. Source data are available online for this figure.

 © The Author(s)

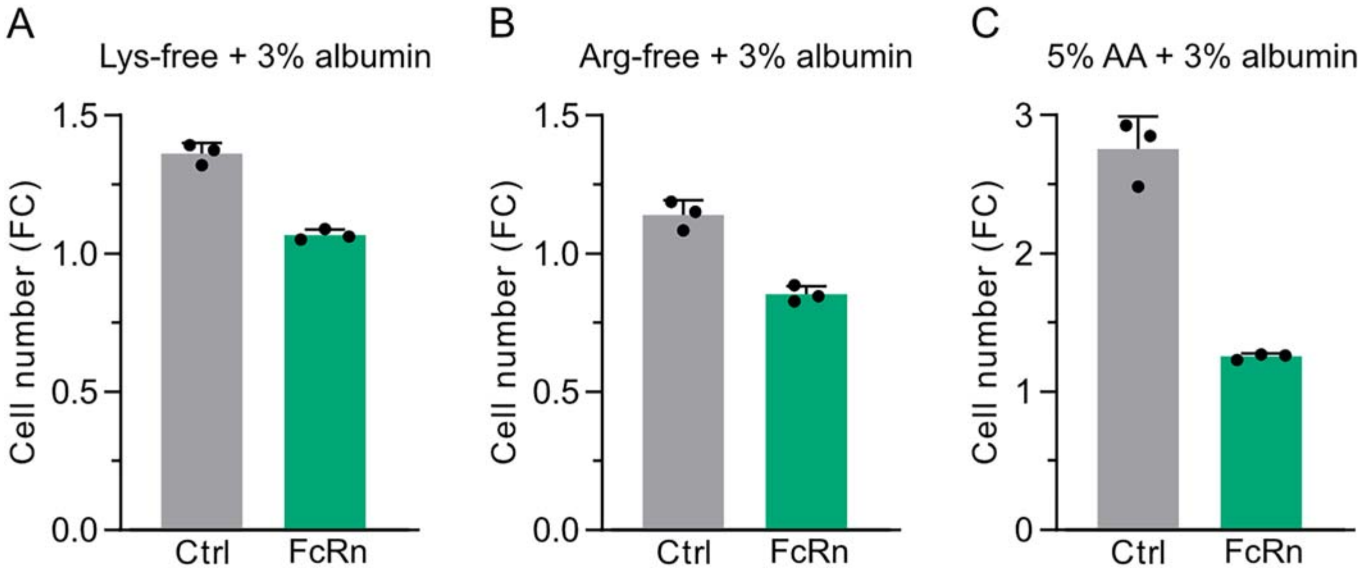

Figure EV4. FcRn suppresses the proliferation of cells that depend on albumin as a nutrient.

(**A–C**) Fold change (FC) in cell number of Kras^{G12D} MEFs after 4 days in amino acid-deficient media supplemented with 3% albumin; (**A**) lysine-free; (**B**) arginine-free; (**C**) all amino acids at 5% of standard medium (DMEM/F-12). Data information: (**A–C**) Data are mean ± SD (three technical replicates from one representative out of $n = 2$–3 independent experiments). Source data are available online for this figure.
