# Supplementary material for: Ras promotes macropinocytic nutrient uptake by suppressing the albumin recycling receptor FcRn
Source: EMBO Rep. 2026 Apr 29;27(12):3214–30. doi: 10.1038/s44319-026-00787-4 (PMC13303908; doi:10.1038/s44319-026-00787-4)
Supplement: Supplementary file 1 — Peer Review File [file 44319_2026_787_MOESM1_ESM.pdf]

EMBO reports

Ras promotes macropinocytic nutrient uptake by suppressing the albumin recycling receptor FcRn

Rafael Paschoal de Campos, Xuxia Wu, Aslihan Inal, Zhao Liu, Craig Thompson, and Wilhelm Palm

Corresponding author(s): Wilhelm Palm (w.palm@dkfz-heidelberg.de)

Review Timeline:		
	Submission Date:	29th Jul 25
	Editorial Decision:	18th Sep 25
	Revision Received:	25th Feb 26
	Editorial Decision:	2nd Apr 26
	Revision Received:	9th Apr 26
	Accepted:	15th Apr 26

Editor: Martina Rembold

Transaction Report:

(Note: With the exception of the correction of typographical or spelling errors that could be a source of ambiguity, letters and reports are not edited. Depending on transfer agreements, referee reports obtained elsewhere may or may not be included in this compilation. Referee reports are anonymous unless the Referee chooses to sign their reports.)

Dear Dr. Palm

Thank you for the submission of your research manuscript to our journal. I apologize for the delay in handling your manuscript, but we have now received the full set of referee reports that is copied below.

As you will see, the referees acknowledge that the findings are interesting and that the conclusions are overall supported by the data presented but they also raise a number of concerns and have suggestions how to further strengthen the data, which should be addressed.

Given these constructive comments, we would like to invite you to revise your manuscript with the understanding that the referee concerns (as detailed above and in their reports) must be fully addressed and their suggestions taken on board. Please address all referee concerns in a complete point-by-point response. Acceptance of the manuscript will depend on a positive outcome of a second round of review. It is EMBO Reports policy to allow a single round of revision only and acceptance or rejection of the manuscript will therefore depend on the completeness of your responses included in the next, final version of the manuscript.

We realize that it is difficult to revise to a specific deadline. In the interest of protecting the conceptual advance provided by the work, we recommend a revision within 3 months (December 19th). Please discuss the revision progress ahead of this time with the editor if you require more time to complete the revisions.

I am also happy to discuss the revision further via e-mail or a video call, if you wish.

You can either publish the study as a short report or as a full article. For short reports, the revised manuscript should not exceed 27,000 characters (including spaces but excluding materials & methods and references) and 5 main plus 5 expanded view figures. The results and discussion sections must further be combined, which will help to shorten the manuscript text by eliminating some redundancy that is inevitable when discussing the same experiments twice. For a normal article there are no length limitations, but it should have more than 5 main figures and the results and discussion sections must be separate. In both cases, the entire materials and methods must be included in the main manuscript file.

================

IMPORTANT NOTE:

We perform an initial quality control of all revised manuscripts before re-review. Your manuscript will FAIL this control and the handling will be delayed IN CASE the following APPLIES:

1) A data availability section providing access to data deposited in public databases is missing. If you have not deposited any data, please add a sentence to the data availability section that explains that.

2) Your manuscript contains statistics and error bars based on n=2. Please use scatter blots in these cases. No statistics should be calculated if n=2.

When submitting your revised manuscript, please carefully review the instructions that follow below. Failure to include requested items will delay the evaluation of your revision.

================

When submitting your revised manuscript, we will require:

1) a .docx formatted version of the manuscript text (including legends for main figures, EV figures and tables). Please make sure that the changes are highlighted to be clearly visible.

2) individual production quality figure files as .eps, .tif, .jpg (one file per figure).
Please download our Figure Preparation Guidelines (figure preparation pdf) from our Author Guidelines pages https://www.embopress.org/page/journal/14693178/authorguide for more info on how to prepare your figures.

3) a .docx formatted letter INCLUDING the reviewers' reports and your detailed point-by-point responses to their comments. As part of the EMBO Press transparent editorial process, the point-by-point response is part of the Review Process File (RPF), which will be published alongside your paper.

4) a complete author checklist, which you can download from our author guidelines (). Please insert information in the checklist

that is also reflected in the manuscript. The completed author checklist will also be part of the RPF.

5) Please note that all corresponding authors are required to supply an ORCID ID for their name upon submission of a revised manuscript (). Please find instructions on how to link your ORCID ID to your account in our manuscript tracking system in our Author guidelines
()

6) We replaced Supplementary Information with Expanded View (EV) Figures and Tables that are collapsible/expandable online. A maximum of 5 EV Figures can be typeset. EV Figures should be cited as 'Figure EV1, Figure EV2" etc... in the text and their respective legends should be included in the main text after the legends of regular figures.

- For the figures that you do NOT wish to display as Expanded View figures, they should be bundled together with their legends in a single PDF file called *Appendix*, which should start with a short Table of Content. Appendix figures should be referred to in the main text as: "Appendix Figure S1, Appendix Figure S2" etc. See detailed instructions regarding expanded view here:

- Additional Tables/Datasets should be labeled and referred to as Table EV1, Dataset EV1, etc. Legends have to be provided in a separate tab in case of .xls files. Alternatively, the legend can be supplied as a separate text file (README) and zipped together with the Table/Dataset file.

7) Before submitting your revision, primary datasets (and computer code, where appropriate) produced in this study need to be deposited in an appropriate public database (see <
https://www.embopress.org/page/journal/14693178/authorguide#dataavailability>).

Please remember to provide a reviewer password if the datasets are not yet public.

The accession numbers and database should be listed in a formal "Data Availability " section (placed after Materials & Method) that follows the model below (see also < https://www.embopress.org/page/journal/14693178/authorguide#dataavailability>). Please note that the Data Availability Section is restricted to new primary data that are part of this study.

Data availability

The datasets (and computer code) produced in this study are available in the following databases:

- RNA-Seq data: Gene Expression Omnibus GSE46843 (https://www.ncbi.nlm.nih.gov/geo/query/acc.cgi?acc=GSE46843)
- [data type]: [name of the resource] [accession number/identifier/doi] ([URL or identifiers.org/DATABASE:ACCESSION])

*** Note - All links should resolve to a page where the data can be accessed. ***

8) At EMBO Press we ask authors to provide source data for the main manuscript figures. You will receive a separate email with instructions for providing source data with your revised manuscript, including how to upload and organize the files.

Additional information on source data and instruction on how to label the files are available

9) The journal requires a statement specifying whether or not authors have competing interests (defined as all potential or actual interests that could be perceived to influence the presentation or interpretation of an article). In case of competing interests, this must be specified in your disclosure statement. Further information: https://www.embopress.org/competing-interests

10) Figure legends and data quantification:
The following points must be specified in each figure legend:

- the name of the statistical test used to generate error bars and P values,
- the EXACT p-values,
- the number (n) of independent experiments (please specify technical or biological replicates) underlying each data point,
- the nature of the bars and error bars (s.d., s.e.m.)

- If the data are obtained from n {less than or equal to} 5, show the individual data points in addition to the SD or SEM.
- If the data are obtained from n {less than or equal to} 2, use scatter blots showing the individual data points.

Discussion of statistical methodology can be reported in the materials and methods section, but figure legends should contain a basic description of n, P and the test applied.

See also the guidelines for figure legend preparation:
https://www.embopress.org/page/journal/14693178/authorguide#figureformat

- Please also include scale bars in all microscopy images and define their size in the legend, not the image.

11) Our journal encourages inclusion of *data citations in the reference list* to directly cite datasets that were re-used and obtained from public databases. Data citations in the article text are distinct from normal bibliographical citations and should directly link to the database records from which the data can be accessed. In the main text, data citations are formatted as follows: "Data ref: Smith et al, 2001" or "Data ref: NCBI Sequence Read Archive PRJNA342805, 2017". In the Reference list, data citations must be labeled with "[DATASET]". A data reference must provide the database name, accession number/identifiers and a resolvable link to the landing page from which the data can be accessed at the end of the reference. Further instructions are available at .

12) All Materials and Methods need to be described in the main text using our 'Structured Methods' format. According to this format, the Methods section includes a Reagents and Tools Table (listing key reagents, experimental models, software and relevant equipment and including their sources and relevant identifiers) followed by a Methods and Protocols section describing the methods, ideally using a step-by-step protocol format. The aim is to facilitate adoption of the methodologies across labs. Please download and fill our Reagents and Tools Table template (.docx), which you can find in our author guidelines: https://www.embopress.org/page/journal/14693178/authorguide#structuredmethods.
When submitting your revised manuscript, please do not include the Reagents and Tools Table in the Methods section of the manuscript but upload it as a separate file choosing the file type "Reagent Table".
An example of a Method paper with Structured Methods can be found here: https://www.embopress.org/doi/10.15252/msb.20178071.

13) As part of the EMBO publication's Transparent Editorial Process, EMBO Reports publishes online a Review Process File to accompany accepted manuscripts. This File will be published in conjunction with your paper and will include the referee reports, your point-by-point response and all pertinent correspondence relating to the manuscript.

You are able to opt out of this by letting the editorial office know (emboreports@embo.org). If you do opt out, the Review Process File link will point to the following statement: "No Review Process File is available with this article, as the authors have chosen not to make the review process public in this case."

We would also welcome the submission of cover suggestions, or motifs to be used by our Graphics Illustrator in designing a cover.

I look forward to seeing a revised form of your manuscript when it is ready and please let me know if you have questions or comments regarding the revision.

Yours sincerely,

Martina Rembold, PhD
Senior Editor
EMBO reports

================

Referee #1:

De Campos et al. reports in this manuscript their finding that Ras-MAPK signaling activity represses FcRn expression, thereby albumin reccyling. They show that constitutively activated KRAS mutants suppress promoter activity, transcript and protein levels of FcRn in multiple cell types, leading to decreased turnover and increased lysosomal degradation of albumin. They went on to show that MEK is the downstream signaling pathway responsible for FcRn downregulation, and PI3K activity is dispensable for this. Furthermore, authors tested whether FcRn expression itself can exacerbate amino acid deprivation-mediated proliferation defects. Overall, this study provides original findings connecting oncogenic Kras and MEK signaling to albumin derived amino acid supply, a critical source of nutrients for transformed cancer cells. However, some points remain to be addressed.

1) Authors show the effect of overexpression of KrasG12V and KrasG12D variants in MEFs on FcRn expression (Figure 1). Since the implications of oncogenic Ras activation is mainly in cancer context, can authors test this experiment in the context of a Kras WT cancer cell line, rather than MEFs? BxPC pancreatic cell line could be a model Kras WT cell line, for example, where KrasG12D overexpression and consequent FcRn expression could be tested.
2) In promoter activity assays, could authors identify binding motifs, when mutated FcRn promoter activity is unaffected? This will serve as an internal control for luciferase-based promoter assays in Figure 2.

3) Authors show that PDGF ligand is sufficient to downregulate FcRn expression in the context of serum deprivation (0.1% FBS). If MEK activity is what is regulating downregulation of FcRn, several ligands must work the same way as PDGF. Can authors provide data for EGF and FGF as well to show generalizability of MEK activation by growth factors ligands besides PDGF.
4) Authors suggest that FcRn expression leads to recycling of albumin, causing less degradation in lysosomes, hence, less amino acid availability in the cells. Can authors provide amino acid level measurement upon KrasG12D or G12V overexpression (for experimental setup shown in Figure 1) as well as FcRn overexpression (as in Figure 5G).
5) In Figure 5 G, authors show Leu deprivation as an amino acid auxotrophy model. Can they include other amino acid deficient media (-Arg, -Met) to show generalizability?

Referee #2:

The authors investigate the regulation of macropinocytosis by the albmin recycling receptor FcRn. They find that Ras via MAPK signaling represses the expression of FcRn, which complements the regulation of via PI3K. The manuscript adds mechanistic understanding the regulation of macropinocytosis.

Figure 1A,B: please refer to Figure EV1C, D for the KRAS_G12V mutant or move Figure EV1 to the main.

Please also treat MEFs with hyperactive KRAS with Sotorasib

Is knockdown of FcRn sufficient to increase macropinocytosis in PI3K mutant breast cancer cell lines?

The authors write "investigating how cancer cells evade FcRn-mediated albumin recycling" However, evade may imply an immune cell involvement. I would suggest overcome or suppress as the verb.

Key findings should be demonstrated in cancer cell lines such as CRC or NSCLC cell lines to complement the patient data

Referee #3:

Overall, this study shows an interesting relationship between RAS activation and FcRn expression levels. The data are in vitro in the main part, relying on overexpression constructs, as well as endogenous Ras mutant expression, but are also supported by previously published datasets showing this relationship is present in patients treated with Adagrasib. The Ras phenomenon was backed up by downstream pathways showing no involvement of Akt, with Braf activation data supporting Ras activity through the Mapk pathway decreases FcRn.

While the study is generally well performed and of general interest, there are many aspects that need to be improved in order to ensure confidence in the data presented. In particular, it appears many experiments are either a single experiment, or technical replicates rather than biological replicate experiments. All data throughout the paper should be performed under more robust biological repeats - and each figure legend should explicitly state the n= for biological repeats rather than technical replicates.

Major points:

1 - The functional data provided are focussed on the amount of albumin in cells, and cell numbers as an output in Figure 5G. It would be interesting to know whether the albumin amino acids can directly contribute to free leucine or directly provide amino acids for protein synthesis. Using a 13C-labelled albumin source could be used with metabolomics and or proteomics to directly show the accumulation of free labelled amino acids, or the incorporation of these labelled amino acids into proteins.

2 - The discussion highlights the potential of Nab-Paclitaxel or other albumin conjugated drugs, which should be examined using their various models with altered FcRn levels to further support their data.

3 - The discussion also mentions that albumin and antibodies are the major plasma proteins, and it would be interesting to see whether providing antibodies instead of albumin can elicit the same effect using their models. This is important showing that it is not an albumin specific finding.

Minor points:

1 - Figure 1B should have more information - it currently seems like a single experiment with 10 fields of view analysed. This experiment should be repeated 3 times and statistical analysis for the differences shown. The text says "significant increase" which is currently not supported by the data.

2 - Figure 1C-E should have RAS blots to show expression levels. Again, no information on the numbers of experiments performed - the data need to be robustly performed in independent experiments and adequately described in the figure legends what exactly was done.

3 - Figure 2E has not x-axis label for the time periods.

4 - Figure 3A, B should have BRAF blot to show expression levels.

5 - Figure 3E-D - are these MEFs BRAFv600e induction?

6 - Figure 4 is only technical repeats, not biological repeats. This needs to be repeated.

7 - Figure 5 again is 10 or 8 fields of view from seemingly a single experiment, while others are 3 technical replicates. Similarly for Fig EV1.

Referee #1:

De Campos et al. reports in this manuscript their finding that Ras-MAPK signaling activity represses FcRn expression, thereby albumin reccyling. They show that constitutively activated KRAS mutants suppress promoter activity, transcript and protein levels of FcRn in multiple cell types, leading to decreased turnover and increased lysosomal degradation of albumin. They went on to show that MEK is the downstream signaling pathway responsible for FcRn downregulation, and PI3K activity is dispensable for this. Furthermore, authors tested whether FcRn expression itself can exacerbate amino acid deprivation-mediated proliferation defects. Overall, this study provides original findings connecting oncogenic Kras and MEK signaling to albumin derived amino acid supply, a critical source of nutrients for transformed cancer cells. However, some points remain to be addressed.

We thank the reviewer for the positive and constructive feedback. We have addressed all points raised by the reviewer as detailed below.

1) Authors show the effect of overexpression of KrasG12V and KrasG12D variants in MEFs on FcRn expression (Figure 1). Since the implications of oncogenic Ras activation is mainly in cancer context, can authors test this experiment in the context of a Kras WT cancer cell line, rather than MEFs? BxPC pancreatic cell line could be a model Kras WT cell line, for example, where KrasG12D overexpression and consequent FcRn expression could be tested.

We agree that our findings concerning FcRn suppression by oncogenic Ras mutations are particularly relevant in the context of cancer. While a direct comparison of Kras wild type and mutant cell lines would in principle be informative, this is challenging for FcRn regulation, because hyperactivation of MAPK signaling, via oncogenic mutations either upstream or downstream of Kras, is prevalent in the carcinoma types used in our study. For example, the BxPC3 cells suggested by the reviewer harbor a Braf ΔL485–P490 mutation, which acts as a driver oncogene in this cell line (PMID: 35804932). To compare oncogenic versus physiological Ras–MAPK signaling in a controlled setting, we therefore examined the effects of defined oncogenic Ras mutations in non-transformed MEFs.

We do acknowledge, however, that a more comprehensive analysis of how oncogenic Kras signaling affects FcRn in cancer cells is important. To address this, we have taken the reverse approach by performing additional experiments with pharmacological inhibitors. In the revised manuscript, we now include data for a pan-Kras inhibitor (BI-2865) in two Kras-mutant cancer cell lines (New Figures 2F, G). In addition, we expanded the panel of cancer cell lines treated with a Mek inhibitor (PD0325901) to include four additional cancer cell lines harboring Kras or Braf mutations (New Figures EV3C–F). In all cases, pharmacological inhibition of Kras–MAPK signaling led to a strong upregulation of FcRn expression, supporting the general relevance of this regulation in cancer cells.

2) In promoter activity assays, could authors identify binding motifs, when mutated FcRn promoter activity is unaffected? This will serve as an internal control for luciferase-based promoter assays in Figure 2.

Following the reviewer's advice, we have performed additional experiments to provide internal controls for our luciferase-based promoter activity assays. First, we generated a promoterless firefly luciferase expression construct, which we lentivirally transduced into MEFs, followed

by G418 selection. Of note, the luciferase and antibiotic resistance marker are expressed from the same transcript via a P2A cleavage site, allowing for selection of cells with basal luciferase expression. Promoterless firefly luciferase was expressed at comparable levels regardless of Ras status (New Figure EV2), in contrast to the FcRn promoter-driven reporter, which was substantially suppressed by Hras^{G12V}. These results confirm that the repressive effect of oncogenic Ras is specific to the FcRn promoter rather than globally affecting firefly luciferase expression. We also assessed luciferase expression in the absence of antibiotic selection by transducing cells at high viral titers. Here, the expression of promoterless firefly luciferase was near background levels, comparable to cells expressing only the Renilla luciferase normalization control (Reviewer 1 – Figure 1).

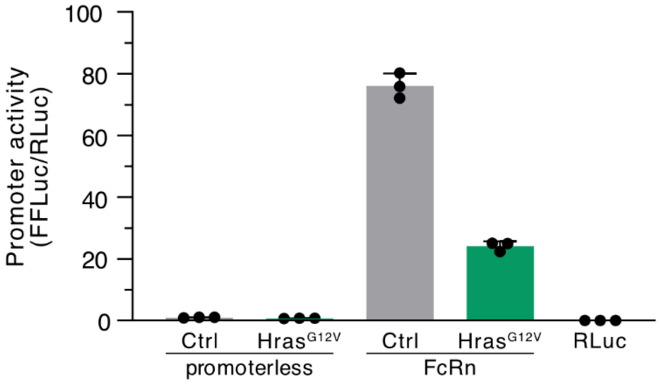

Reviewer 1 - Figure 1 | FcRn promoter activity responds to Ras signaling. Relative luciferase expression in MEFs after 16 h induction of Hras^{G12V}, analyzed with dual-luciferase reporter assay (Firefly luciferase: FFLuc; Renilla luciferase: RLuc). FFluc (under control of the FcRn promoter or promoterless) was transduced at a high viral titer to ensure near-complete infection of the cell population and thus obviate the need for antibiotic selection. Note that FcRn promoter-driven FFluc expression is suppressed by Hras^{G12V}, whereas promoterless FFluc expression is close to background signal (RRluc). Experiments were performed in 0.1% FBS.

Regarding the identification of specific binding motifs, we agree that this is an interesting direction. However, pinpointing the exact repressive elements would first require identifying the specific transcription factor(s) involved and mapping their binding sites. Such a detailed dissection of the transcriptional mechanism of FcRn suppression represents a new line of experiments that is beyond the scope of the present study, which focuses on the signaling pathway and metabolic impact of Ras-mediated FcRn repression.

3) Authors show that PDGF ligand is sufficient to downregulate FcRn expression in the context of serum deprivation (0.1% FBS). If MEK activity is what is regulating downregulation of FcRn, several ligands must work the same way as PDGF. Can authors provide data for EGF and FGF as well to show generalizability of MEK activation by growth factors ligands besides PDGF.

Examining additional growth factors is an excellent suggestion to address the generalizability of our findings. In the revised manuscript, we show that supplementing serum-deficient medium (0.1% FBS) with FGF strongly suppressed FcRn mRNA and protein levels in wild-type MEFs, comparable to the effects of PDGF (New Figures 4G, H). By contrast, EGF had no effect in MEFs, consistent with the low expression levels of the EGF receptor in fibroblasts (Reviewer 1 - Figure 2A–C). In pancreatic cancer KRPC cells, however, which are epithelial-derived, FcRn expression was decreased by EGF (Reviewer 1 - Figure 2D). Together, these results show that FcRn expression is suppressed by a range of related growth factors.

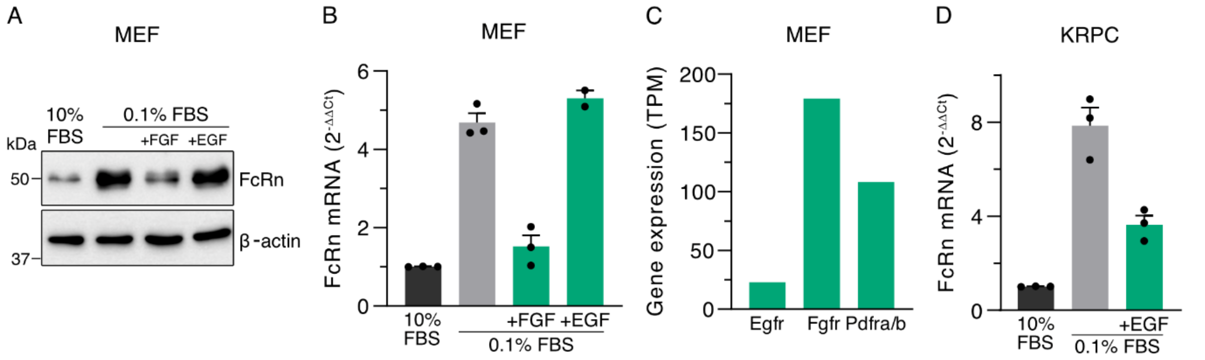

Reviewer 1 - Figure 2 | FcRn expression is suppressed by growth factors. (A, B) FcRn regulation in wild-type MEFs after 16 h in 10% FBS or 0.1% FBS ± FGF or EGF [50 ng/ml]; (A) FcRn protein abundance, analyzed by immunoblotting; (B) relative FcRn mRNA abundance, analyzed by qPCR. (C) Expression levels of different growth factor receptors in MEFs, quantified by RNA-seq. (D) Relative FcRn mRNA abundance in pancreatic cancer KRPC cells after 16 h in 10% FBS or 0.1% FBS ± EGF [50 ng/ml], analyzed by qPCR.

4) Authors suggest that FcRn expression leads to recycling of albumin, causing less degradation in lysosomes, hence, less amino acid availability in the cells. Can authors provide amino acid level measurement upon KrasG12D or G12V overexpression (for experimental setup shown in Figure 1) as well as FcRn overexpression (as in Figure 5G).

Following the reviewer's advice, we have quantified intracellular amino acid levels with LC-MS, focusing on essential amino acids as these must be acquired from exogenous sources. We analyzed Ras-mutant MEFs and KRPC cells under culture conditions matching those in Figures 1 and 5 (supplemented with 3% BSA). Under these conditions, intracellular amino acid levels did not consistently change across the different genetic manipulations (induction of oncogenic Ras or ectopic FcRn expression, Reviewer 1 – Figure 3). These results are consistent with established metabolic principles and previous observations. In complete medium (Figure 1), free amino acids are provided at very high levels, which likely mask the effect of albumin catabolism. In leucine-free medium (Figure 5), leucine generated through lysosomal albumin catabolism is expected to be rapidly used for protein synthesis rather than accumulating in the free pool. Accordingly, cell growth (net increase in protein mass) is widely used as a sensitive and physiologically relevant proxy for amino acid acquisition from extracellular protein (e.g., PMID: 26144316, 36074822, 39627191). Thus, while LC-MS analysis did not reveal changes in intracellular amino acid levels, the proliferation assays in Figure 5 demonstrate the functional impact of FcRn on the nutritional utilization of albumin.

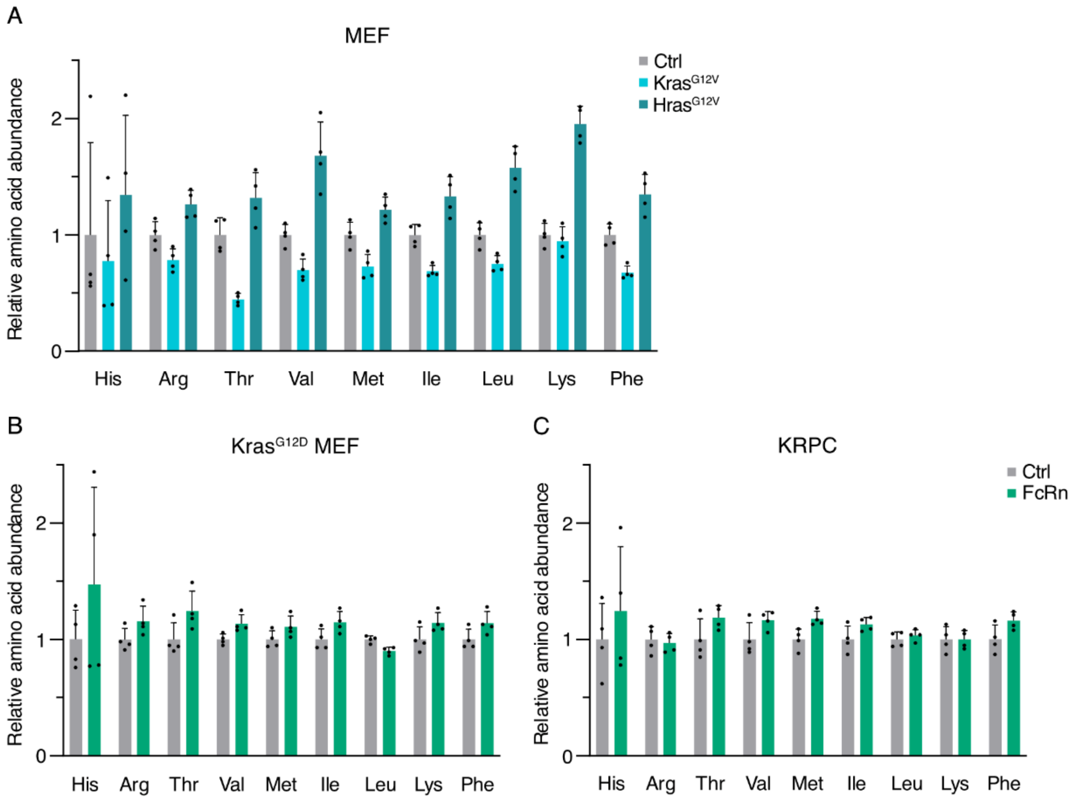

Reviewer 1 - Figure 3 | Quantification of intracellular amino acid levels in response to Ras signaling or FcRn expression. Intracellular amino acid levels were quantified by liquid chromatography-mass spectrometry (LC-MS). (A) MEFs after 24 h Kras^{G12V} or Hras^{G12V} induction in 0.1% FBS + 3% BSA. (B) Kras^{G12D} MEFs after 24 h in leucine-free medium + 10% dialyzed FBS, 3% BSA. (C) KRPC cells after 24 h in leucine-free medium + 10% dialyzed FBS, 3% BSA.

5) In Figure 5 G, authors show Leu deprivation as an amino acid auxotrophy model. Can they include other amino acid deficient media (-Arg, -Met) to show generalizability?

We thank the referee for this suggestion. We typically utilize leucine-free medium for albumin-related growth experiments because leucine is highly abundant in both the mammalian proteome and albumin, and its primary metabolic fate is incorporation into proteins. To demonstrate the generalizability of our findings, we have now conducted similar assays with Kras^{G12D} MEFs in media deficient for arginine or lysine. Furthermore, to provide a more physiological context, we included a low amino acid condition in which all amino acids are provided at 5% of their standard concentrations. In all cases, the media were supplemented with 3% BSA as an alternative amino acid source. Ectopic FcRn expression reduced cell proliferation/viability across these amino acid-deficient conditions (New Figure EV4).

Referee #2:

The authors investigate the regulation of macropinocytosis by the albmin recycling receptor FcRn. They find that Ras via MAPK signaling represses the expression of FcRn, which complements the regulation of via PI3K. The manuscript adds mechanistic understanding the regulation of macropinocytosis.

We thank the reviewer for the positive and constructive feedback and for raising several points to improve the manuscript, which we have addressed as detailed below.

Figure 1A,B: please refer to Figure EV1C, D for the KRAS_G12V mutant or move Figure EV1 to the main.

Thanks for this suggestion. We now cite Figures EV1A and B (formerly EV1C and D) concerning the Kras^{G12V} mutation in the results section discussing Figures 1A and B.

Please also treat MEFs with hyperactive KRAS with Sotorasib

Examining the effect of pharmacological inhibition of Kras in MEFs is an excellent idea. Sotorasib is a selective inhibitor of the G12C mutation, however, and thus not active against the G12D and G12V mutations we expressed in MEFs. To test Kras inhibition in our MEF lines, we instead utilized BI-2865, a pan-Kras inhibitor that prevents the activation of a broad range of Kras mutants (PMID: 37258666). Indeed, treating Kras^{G12V}-expressing MEFs with BI-2865 led to a substantial increase in FcRn protein abundance (New Figure 1F).

Is knockdown of FcRn sufficient to increase macropinocytosis in PI3K mutant breast cancer cell lines?

Following the reviewer's advice, we used CRISPR/Cas9 to deplete FcRn in T47D, a breast cancer cell line harboring the PIK3CA H1047R mutation. To assess the effect on macropinocytosis, we monitored uptake of fluorescently labeled 70 kDa dextran, which is selectively internalized through macropinosomes. We also included 10 kDa dextran as a general marker for fluid-phase endocytosis. Dextran uptake was comparable between FcRn knockout and control cells, indicating that FcRn does not affect macropinocytosis (Reviewer 2 – Figure 1). These results are consistent with the function of FcRn in endosomal recycling of albumin rather than macropinocytic uptake.

The authors write "investigating how cancer cells evade FcRn-mediated albumin recycling" However, evade may imply an immune cell involvement. I would suggest overcome or suppress as the verb.

Good point, thanks for spotting this. We have changed 'evade' to 'overcome'.

Key findings should be demonstrated in cancer cell lines such as CRC or NSCLC cell lines to complement the patient data.

We thank the reviewer for raising this point. In the revised manuscript, we now include data showing that the pan-Kras inhibitor BI-2865 increases FcRn expression in DLD-1 cells (colorectal cancer, $Kras^{G13D}$) and lung KP cells (non-small cell lung cancer, $Kras^{G12D}$) (New Figures 2F, G). Moreover, we demonstrate that FcRn expression increases upon treatment with the Mek inhibitor PD0325901 in various cell lines, including DLD-1, lung KP, SW1417 (colorectal cancer, $Braf^{V600E}$) and RKO (colorectal cancer, $Braf^{V600E}$) (New Figures EV3C–F). We observe comparable effects of Kras–MAPK pathway inhibition in the colorectal and lung cancer cell lines, in pancreatic cancer cells and in MEFs transformed with oncogenic Kras or Braf variants. Altogether, these results indicate that FcRn expression is suppressed by oncogenic Kras – MAPK signaling across a range of different cancer types harboring activating mutations in this signaling pathway.

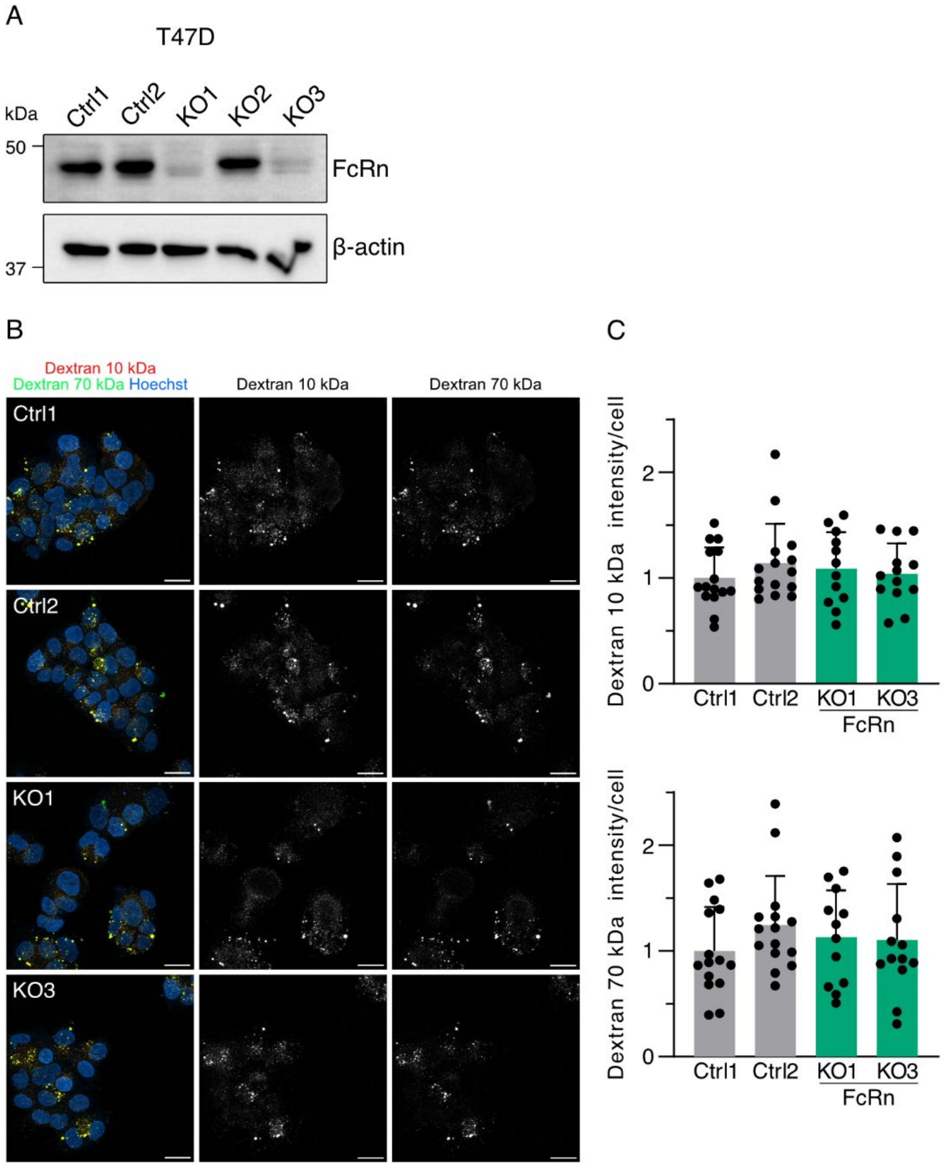

Reviewer 2 - Figure 1 | FcRn depletion does not affect macropinocytosis in T47D cells. (A) FcRn levels in T47D cells transduced with three distinct sgRNAs (KO1–3) and two control sgRNAs (Ctrl1, 2), analyzed by immunoblotting. sgRNAs 1 and 3 were selected for further experiments due to high knockout efficiency. (B) Intracellular levels of fluorescently labeled 10 kDa dextran and 70 kDa dextran after 1 h uptake. Scale bars = 20 µm. (C) Quantification of dextran fluorescence in cells shown in (B).

Referee #3:

Overall, this study shows an interesting relationship between RAS activation and FcRn expression levels. The data are in vitro in the main part, relying on overexpression constructs, as well as endogenous Ras mutant expression, but are also supported by previously published datasets showing this relationship is present in patients treated with Adagrasib. The Ras phenomenon was backed up by downstream pathways showing no involvement of Akt, with Braf activation data supporting Ras activity through the Mapk pathway decreases FcRn.

While the study is generally well performed and of general interest, there are many aspects that need to be improved in order to ensure confidence in the data presented. In particular, it appears many experiments are either a single experiment, or technical replicates rather than biological replicate experiments. All data throughout the paper should be performed under more robust biological repeats - and each figure legend should explicitly state the n= for biological repeats rather than technical replicates.

We thank the reviewer for highlighting our study as generally well-performed and of interest, and we appreciate the constructive and detailed feedback. In the original manuscript, all data presented reflected multiple independent biological replicates; we apologize for not having indicated this adequately. To address the reviewer's concern and to ensure transparency and robustness, we have thoroughly revised the data presentation and figure legends throughout the manuscript:

- We now present imaging data as superplots, displaying the means of biologically independent experiments alongside individual fields of view for Figures 1B, 5B and 5E.

- We have updated the presentation of qPCR data to provide replicate means of biologically independent experiments for Figures 2A, B, C, D, E, F, G; Figure 3C, F, K; Figure 4D, H; and Figure EV3C, D, E, F.

- Each figure legend now includes a 'Data information' section that defines the number of biological replicates and where applicable the nature of technical replicates and statistical tests used.

Finally, we would like to emphasize that our study design—in addition to independent replication of the same experiment—ensures robustness of our findings through systematic orthogonal experimentation, which we have further expanded in the revised manuscript. We interrogate signaling pathways using independent genetic manipulations (expression of oncogenic Kras, Hras and Braf variants) pharmacological approaches (inhibition of Kras, Mek and Erk) and physiological activators (FGF and PDGF). Furthermore, we confirm our findings across multiple murine and human cell lines using three distinct readouts for FcRn regulation (protein abundance, mRNA levels, and promoter activity) and several growth conditions (w/o Leu, Lys or Arg, 5 % total amino acids). These diverse approaches consistently corroborate the key conclusion of our manuscript that FcRn expression is suppressed through the Ras–MAPK signaling pathway. In addition to these general improvements to the manuscript, we have addressed the specific points raised by the reviewer as detailed below.

Major points:

1 - The functional data provided are focussed on the amount of albumin in cells, and cell numbers as an output in Figure 5G. It would be interesting to know whether the albumin amino acids can directly contribute to free leucine or directly provide amino acids for protein synthesis. Using a 13C-labelled albumin source could be used with metabolomics and or proteomics to directly show the accumulation of free labelled amino acids, or the incorporation of these labelled amino acids into proteins.

We appreciate the reviewer's suggestion to conduct isotope tracing with ^{13}C-labelled albumin, which is an interesting but very challenging experiment. Such isotope-labeled albumin is commercially only available in minute quantities as a mass spectrometry standard and prohibitively expensive for cell culture feeding experiments. Alternatively, producing cell culture-grade albumin in sufficient quantities would require establishing a complex expression and purification system, which represents a significant undertaking. Importantly, the use of cell growth and the associated net increase in protein mass as a proxy for amino acid acquisition from extracellular protein is a well-established and validated approach in the field of cancer metabolism. We and others have demonstrated that cell growth in media lacking essential amino acids but supplemented with physiological albumin levels reliably reflects amino acid production through its lysosomal proteolysis (e.g., PMID: 26144316, 36074822, 39627191), making it well-suited to investigate the impact of FcRn. Performing the suggested isotope-tracing experiment would thus unlikely represent a significant advance concerning our main conclusion that FcRn suppresses the nutritional utilization of albumin.

2 - The discussion highlights the potential of Nab-Paclitaxel or other albumin conjugated drugs, which should be examined using their various models with altered FcRn levels to further support their data.

We thank the reviewer for raising this point. Based on our findings concerning FcRn suppression by Ras and previous results implicating FcRn levels in the efficacy of albumin-conjugated chemotherapeutics (PMID: 30653981), we consider a brief discussion of the potential implications for Ras-driven cancers of interest. Addressing this experimentally, however, would represent a distinct pharmacological study rather than a mechanistic investigation of FcRn regulation. Such experiments are beyond the scope of our current work and are not required to support our central conclusions. We have revised the discussion to more clearly distinguish between experimentally validated results and potential therapeutic implications.

3 - The discussion also mentions that albumin and antibodies are the major plasma proteins, and it would be interesting to see whether providing antibodies instead of albumin can elicit the same effect using their models. This is important showing that it is not an albumin specific finding.

We agree that exploring the nutritional use of immunoglobulin G (IgG), the other principal FcRn cargo, would be interesting. Whether IgG serves as a meaningful amino acid source in cancer, however, has not been addressed yet and would first need to be addressed to provide a framework in which to study the potential impact of FcRn regulation. This is beyond the scope of our manuscript, which focuses on the known role of albumin as a nutrient source in Ras-

driven cancers. We have revised the discussion to clarify that whereas the nutritional importance of albumin in cancer is established, a corresponding role for immunoglobulins remains speculative. We respectfully point out that our study specifically addresses the regulation and metabolic relevance of FcRn with regard to its major cargo, albumin—by definition, our findings are therefore albumin-specific. Given that albumin represents the major source of non-cellular biomass in circulation, our findings provide fundamental insights into metabolic dysregulation by oncogenic Ras, regardless of a potential impact of quantitatively lesser FcRn cargoes.

Minor points:

1 - Figure 1B should have more information - it currently seems like a single experiment with 10 fields of view analysed. This experiment should be repeated 3 times and statistical analysis for the differences shown. The text says "significant increase" which is currently not supported by the data.

We thank the reviewer for the suggestion to improve the robustness of our imaging data. We have replaced Figure 1B with a superplot overlaying the replicate means of three biologically independent experiments and individual fields of view from all experiments. We have also added statistical analysis based on the replicate means, demonstrating a significant effect. These findings corroborate previous reports by us and others of increased albumin degradation in Ras-transformed cells (e.g., PMIDs: 26144316, 23665962) within the experimental context of the present study.

2 - Figure 1C-E should have RAS blots to show expression levels. Again, no information on the numbers of experiments performed - the data need to be robustly performed in independent experiments and adequately described in the figure legends what exactly was done.

We have added immunoblots showing Ras protein levels for Figures 1C, D and F, which depict cell lines stably expressing Ras mutants via viral transduction. We did not include Ras immunoblots for Figure 1E (formerly 1D); this experiment uses mouse embryonic fibroblasts in which wild-type Kras or Kras^{G12D} is expressed from the endogenous Kras locus, a widely used genetic system to study the G12D mutation (see e.g., PMID: 15093544, 26144316). We apologize for the lack of replicate information in the original manuscript and now provide this in a new 'Data information' section in all figure legends. Additionally, we have expanded the Methods section to describe the different ectopic Ras expression systems.

3 - Figure 2E has not x-axis label for the time periods.

Thanks for spotting this! We added the x-axis label to Figure 2H (formerly 2E).

4 - Figure 3A, B should have BRAF blot to show expression levels.

We have added immunoblots showing Braf expression levels to Figure 3B, which depicts MEFs with ectopic expression of Braf^{V600E}. In Figure 3A, the lanes show protein levels from Hras^{G12V}-expressing MEFs treated with inhibitors against PI3-kinase or Akt. Because this figure

addresses the putative role of PI3-kinase signaling in FcRn expression, we have not included blots for Braf, which functions in a distinct signaling pathway.

5 - Figure 3E-D - are these MEFs BRAFv600e induction?

We apologize for this omission. The MEFs in Figures 3E–G express $Hras^{G12V}$, which we now explain in the revised legend.

6 - Figure 4 is only technical repeats, not biological repeats. This needs to be repeated.

We have replaced the RT-qPCR results in Figures 4D and H with plots showing replicate means from three biologically independent experiments. We have also added statistical analyses demonstrating significant effects.

7 - Figure 5 again is 10 or 8 fields of view from seemingly a single experiment, while others are 3 technical replicates. Similarly for Fig EV1.

We have replaced Figures 5B and 5E with superplots overlaying the replicate means of three biologically independent experiments and the individual fields of view. We have also added statistical analysis demonstrating significant effects for both albumin uptake and degradation. Figure EV1 is a control to reproduce previous findings (PMID: 31649109) in our experimental context; we respectfully suggest that the current presentation of these supplementary control data is appropriate.

1st Revision - Editorial Decision 2nd Apr 2026

Dear Wilhelm,

Thank you for the submission of your revised manuscript to EMBO reports. We have now received the full set of referee reports that is copied below.

As you will see, all referees are very positive about the study and recommend publication. Before I can officially accept the manuscript, I need you to address some minor points below:

- Your manuscript will be published in our Reports section. This requires a combined "Results and Discussion" section. Please also remove the header "Concluding remarks".

- Materials and Methods should be Methods

- The Data Availability section needs to be moved before the Acknowledgments

- Regarding the Author Contributions, we now use CRediT to specify the contributions of each author in the journal submission system. CRediT replaces the author contribution section, which therefore needs to be removed from the manuscript text. You can use the free text box in our system if you wish to provide more detailed descriptions. See also guide to authors https://www.embopress.org/page/journal/14693178/authorguide#authorshipguidelines.

- The Reagents and Tools table needs to be removed from the manuscript and uploaded as a separate .docx file.

- You state in the methods section:
"Gene expression data from non-small cell lung cancer (NSCLC) and colorectal cancer (CRC) patients before and 8 days after treatment with the KrasG12C-selective inhibitor adagrasib were obtained from a previously published dataset (Klomp et al., 2024)."

Here you could introduce a Data citation. You first cite the paper and then the Dataset itself. In the tex this looks like: (Klomp et al., 2024; data ref: Klomp et al., 2024).
In the reference list you add the data citation, which includes in esssence the same authors but instead of referring to the paper you add the URL that resolves directly to the dataset and add [DATASET] at the end. Please see the relevant section in our Author Guidelines.

- Author Checklist please select a response from the pulldown menu for cells D81 and D84.

- Table EV1 is called out but missing from the files. I assume you meant to call out Appendix Table S1? You could actually upload this table as Table EV1 to avoid having one Supplementary PDF only for the table. Alternatively, you could incorporate this short table as Table 1 in the Methods section. I would recommend the latter.

- Source Data: the immunofluorescent images would be more useful if supplied as original files or at least as a stack/composite that can be split into individual channels in Fiji or ImageJ.

- We screen numerical data for duplications using a custom-made program. All looks fine but the quantification provided for Figure EV3a shows a duplication for two numbers with exactly the same up to the 6th decimal. Can you please double-check these? Color-coded file is attached.

- Finally, EMBO Reports papers are accompanied online by
A) a short (1-2 sentences) summary of the findings and their significance,
B) 2-3 bullet points highlighting key results and
C) a schematic summary figure that provides a sketch of the major findings (not a data image).
Please provide the summary figure as a separate file in PNG or JPG format at a size of 550x300 pixels (width x height). Please note that the size is rather small and that text needs to be readable at the final size. Please send us this information along with the revised manuscript.

We look forward to seeing a final version of your manuscript as soon as possible.

With kind regards,

Martina

Martina Rembold, PhD
Senior Editor
EMBO reports

=================

Referee #1:

The authors addressed my points.

Referee #2:

the authors addressed my comments

Referee #3:

The authors have adequately revised the manuscript.

All minor editorial requests have been addressed by the authors.

Dr. Wilhelm Palm
German Cancer Research Center (DKFZ)
Cell Biology and Tumor Biology
Im Neuenheimer Feld 280
Heidelberg, Baden-Württemberg 69120
Germany

Dear Wilhelm,

I am very pleased to accept your manuscript for publication in the next available issue of EMBO reports. Thank you for your contribution to our journal.

Your manuscript will be processed for publication by EMBO Press. It will be copy edited and you will receive page proofs prior to publication. Please note that you will be contacted by Springer Nature Author Services to complete licensing and payment information.

You may qualify for financial assistance for your publication charges - either via a Springer Nature fully open access agreement or an EMBO initiative. Check your eligibility: https://link.springer.com/journal/44319/how-to-publish-with-us

Should you be planning a Press Release on your article, please get in contact with embo_production@springernature.com as early as possible in order to coordinate publication and release dates.

If you have any questions, please do not hesitate to contact the Editorial Office. Thank you for your contribution to EMBO Reports.

Kind regards,

Martina

Martina Rembold, PhD
Senior Editor
EMBO reports

>>> Please note that it is EMBO Reports policy for the transcript of the editorial process (containing referee reports and your response letter) to be published as an online supplement to each paper. If you do NOT want this, you will need to inform the Editorial Office via email immediately. More information is available here: https://link.springer.com/partners/embo-press/editorial-policies#Peer%20review

EMBO Press Author Checklist

Corresponding Author Name: Wilhelm Palm
Journal Submitted to: EMBO Reports
Manuscript Number: EMBOR-2025-62422V1

USEFUL LINKS FOR COMPLETING THIS FORM
The EMBO Journal - Author Guidelines
EMBO Reports - Author Guidelines
Molecular Systems Biology - Author Guidelines
EMBO Molecular Medicine - Author Guidelines

Reporting Checklist for Life Science Articles (updated January
This checklist is adapted from Materials Design Analysis Reporting (MDAR) Checklist for Authors. MDAR establishes a minimum set of requirements in transparent reporting in the life sciences (see Statement of Task: 10.31222/osf.io/9sm4x). Please follow the journal's guidelines in preparing your
Please note that a copy of this checklist will be published alongside your article.

Abridged guidelines for figures
1. Data
The data shown in figures should satisfy the following conditions:
- the data were obtained and processed according to the field's best practice and are presented to reflect the results of the experiments in an accurate and unbiased manner.
- ideally, figure panels should include only measurements that are directly comparable to each other and obtained with the same assay.
- plots include clearly labeled error bars for independent experiments and sample sizes. Unless justified, error bars should not be shown for technical
- if n<5, the individual data points from each experiment should be plotted. Any statistical test employed should be justified.
- Source Data should be included to report the data underlying figures according to the guidelines set out in the authorship guidelines on Data

2. Captions
Each figure caption should contain the following information, for each panel where they are relevant:
- a specification of the experimental system investigated (eg cell line, species name).
- the assay(s) and method(s) used to carry out the reported observations and measurements.
- an explicit mention of the biological and chemical entity(ies) that are being measured.
- an explicit mention of the biological and chemical entity(ies) that are altered/varied/perturbed in a controlled manner.
- the exact sample size (n) for each experimental group/condition, given as a number, not a range;
- a description of the sample collection allowing the reader to understand whether the samples represent technical or biological replicates (including how many animals, litters, cultures, etc.).
- a statement of how many times the experiment shown was independently replicated in the laboratory.
- definitions of statistical methods and measures:
  - common tests, such as t-test (please specify whether paired vs. unpaired), simple χ2 tests, Wilcoxon and Mann-Whitney tests, can be unambiguously identified by name only, but more complex techniques should be described in the methods section;
  - are tests one-sided or two-sided?
  - are there adjustments for multiple comparisons?
  - exact statistical test results, e.g., P values = x but not P values < x;
  - definition of 'center values' as median or average;
  - definition of error bars as s.d. or s.e.m.

Please complete ALL of the questions below.
Select "Not Applicable" only when the requested information is not relevant for your study.

Materials

Newly Created Materials	Information included in the manuscript?	In which section is the information available? (Reagents and Tools Table, Materials and Methods, Figures, Data Availability Section)
New materials and reagents need to be available; do any restrictions apply?	Yes	Materials and Methods

Antibodies	Information included in the manuscript?	In which section is the information available? (Reagents and Tools Table, Materials and Methods, Figures, Data Availability Section)
For **antibodies** provide the following information:<br>- Commercial antibodies: RRID (if possible) or supplier name, catalogue number and or/clone number<br>- Non-commercial: RRID or citation	Yes	Materials and Methods

DNA and RNA sequences	Information included in the manuscript?	In which section is the information available? (Reagents and Tools Table, Materials and Methods, Figures, Data Availability Section)
**Short novel DNA or RNA including primers, probes**: provide the sequences.	Yes	Appendix

Cell materials	Information included in the manuscript?	In which section is the information available? (Reagents and Tools Table, Materials and Methods, Figures, Data Availability Section)
**Cell lines:** Provide species information, strain. Provide accession number in repository **OR** supplier name, catalog number, clone number, and/**OR** RRID.	Yes	Materials and Methods
**Primary cultures:** Provide species, strain, sex of origin, genetic modification status.	Not Applicable	
Report if the cell lines were recently **authenticated** (e.g., by STR profiling) and tested for mycoplasma contamination.	Yes	Materials and Methods

Experimental animals	Information included in the manuscript?	In which section is the information available? (Reagents and Tools Table, Materials and Methods, Figures, Data Availability Section)
**Laboratory animals or Model organisms:** Provide species, strain, sex, age, genetic modification status. Provide accession number in repository **OR** supplier name, catalog number, clone number, **OR** RRID.	Not Applicable	
**Animal observed in or captured from the field:** Provide species, sex, and age where possible.	Not Applicable	
Please detail **housing and husbandry conditions.**	Not Applicable	

Plants and microbes	Information included in the manuscript?	In which section is the information available? (Reagents and Tools Table, Materials and Methods, Figures, Data Availability Section)
**Plants:** provide species and strain, ecotype and cultivar where relevant, unique accession number if available, and source (including location for collected wild specimens).	Not Applicable	
**Microbes:** provide species and strain, unique accession number if available, and source.	Not Applicable	

Human research participants	Information included in the manuscript?	In which section is the information available? (Reagents and Tools Table, Materials and Methods, Figures, Data Availability Section)
If collected and within the bounds of privacy constraints report on age, sex and gender or ethnicity for all study participants.	Not Applicable	

Core facilities	Information included in the manuscript?	In which section is the information available? (Reagents and Tools Table, Materials and Methods, Figures, Data Availability Section)
If your work benefited from core facilities, was their service mentioned in the acknowledgments section?	Yes	Acknowledgements

Design

Study protocol	Information included in the manuscript?	In which section is the information available? (Reagents and Tools Table, Materials and Methods, Figures, Data Availability Section)
If study protocol has been **pre-registered, provide DOI in the manuscript**. For clinical trials, provide the trial registration number **OR** cite DOI.	Not Applicable	
Report the **clinical trial registration number** (at ClinicalTrials.gov or equivalent), where applicable.	Not Applicable	

Laboratory protocol	Information included in the manuscript?	In which section is the information available? (Reagents and Tools Table, Materials and Methods, Figures, Data Availability Section)
Provide DOI OR other citation details if **external detailed step-by-step protocols** are available.	Not Applicable	

Experimental study design and statistics	Information included in the manuscript?	In which section is the information available? (Reagents and Tools Table, Materials and Methods, Figures, Data Availability Section)
Include a statement about **sample size** estimate even if no statistical methods were used.	Yes	Figures
Were any steps taken to minimize the effects of subjective bias when allocating animals/samples to treatment (e.g. **randomization procedure**)? If yes, have they been described?	Yes	For imaging experiments, images were taken in a random manner across the entire sample; no randomization procedure was used for other experiments.
Include a statement about **blinding** even if no blinding was done.	Not Applicable	
Describe **inclusion/exclusion criteria** if samples or animals were excluded from the analysis. Were the criteria pre-established?<br><br>If sample or data points were omitted from analysis, report if this was due to attrition or intentional exclusion and provide justification.	Not Applicable	
For every figure, are **statistical tests** justified as appropriate? Do the data meet the assumptions of the tests (e.g., normal distribution)? Describe any methods used to assess it. Is there an estimate of variation within each group of data? Is the variance similar between the groups that are being statistically compared?	Yes	Statistical tests were performed on data representing independent biological experiments. Two-tailed unpaired t-test with Welch correction were used to correct for the differences in variance between experimental groups.

Sample definition and in-laboratory replication	Information included in the manuscript?	In which section is the information available? (Reagents and Tools Table, Materials and Methods, Figures, Data Availability Section)
In the figure legends: state number of times the experiment was **replicated** in laboratory.	Yes	Figures
In the figure legends: define whether data describe **technical or biological replicates**.	Yes	Figures

Ethics

Ethics	Information included in the manuscript?	In which section is the information available? (Reagents and Tools Table, Materials and Methods, Figures, Data Availability Section)
Studies involving **human participants**: State details of **authority granting ethics approval** (IRB or equivalent committee(s), provide reference number for approval.	Not Applicable	
Studies involving **human participants**: Include a statement confirming that **informed consent** was obtained from all subjects and that the experiments conformed to the principles set out in the WMA Declaration of Helsinki and the Department of Health and Human Services Belmont Report.	Not Applicable	
Studies involving **human participants:** For publication of **patient photos**, include a statement confirming that consent to publish was obtained.	Not Applicable	
Studies involving experimental **animals**: State details of **authority granting ethics approval** (IRB or equivalent committee(s), provide reference number for approval. Include a statement of compliance with ethical regulations.	Not Applicable	
Studies involving **specimen and field samples:** State if relevant **permits** obtained, provide details of authority approving study; if none were required, explain why.	Not Applicable	

Dual Use Research of Concern (DURC)	Information included in the manuscript?	In which section is the information available? (Reagents and Tools Table, Materials and Methods, Figures, Data Availability Section)
Could your study fall under dual use research restrictions? Please check biosecurity documents and list of **select agents and toxins** (CDC): https://www.selectagents.gov/sat/list.htm	Not Applicable	
If you used a select agent, is the security level of the lab appropriate and reported in the manuscript?	Not Applicable	
If a study is subject to dual use research of concern regulations, is the name of the **authority granting approval and reference number** for the regulatory approval provided in the manuscript?	Not Applicable	

Reporting

The MDAR framework recommends adoption of discipline-specific guidelines, established and endorsed through community initiatives. Journals have their own policy about requiring specific guidelines and recommendations to complement MDAR.

Adherence to community standards	Information included in the manuscript?	In which section is the information available? (Reagents and Tools Table, Materials and Methods, Figures, Data Availability Section)
State if relevant guidelines or checklists (e.g., **ICMJE, MIBBI, ARRIVE, PRISMA**) have been followed or provided.	Not Applicable	
For **tumor marker prognostic studies,** we recommend that you follow the **REMARK** reporting guidelines (see link list at top right). See author guidelines, under 'Reporting Guidelines'. Please confirm you have followed these guidelines.	Not Applicable	
For **phase II and III randomized controlled trials**, please refer to the **CONSORT** flow diagram (see link list at top right) and submit the CONSORT checklist (see link list at top right) with your submission. See author guidelines, under 'Reporting Guidelines'. Please confirm you have submitted this list.	Not Applicable	

Data Availability

Data availability	Information included in the manuscript?	In which section is the information available? (Reagents and Tools Table, Materials and Methods, Figures, Data Availability Section)
Have **primary datasets** been deposited according to the journal's guidelines (see 'Data Deposition' section) and the respective accession numbers provided in the Data Availability Section?	Not Applicable	
Were **human clinical and genomic datasets** deposited in a public access-controlled repository in accordance to ethical obligations to the patients and to the applicable consent agreement?	Not Applicable	
Are **computational models** that are central and integral to a study available without restrictions in a machine-readable form? Were the relevant accession numbers or links provided?	Not Applicable	
If publicly available data were reused, provide the respective **data citations in the reference list**.	Yes	Materials and Methods, Figures, References
